# Autologous dendritic cell vaccination against HIV-1 induces changes in natural killer cell phenotype and functionality

**DOI:** 10.1038/s41541-023-00631-z

**Published:** 2023-03-02

**Authors:** Thessa Laeremans, Sabine den Roover, Cynthia Lungu, Sigrid D’haese, Rob A. Gruters, Sabine D. Allard, Joeri L. Aerts

**Affiliations:** 1 Neuro-Aging and Viro-Immunotherapy Research Group, Vrije Universiteit Brussel, Brussels, Belgium; 2 Department of Viroscience, Erasmus Medical Center, Rotterdam, The Netherlands; 3 Department of Internal Medicine and Infectious Diseases, Universitair Ziekenhuis Brussel and Vrije Universiteit Brussel, Brussels, Belgium

**Keywords:** HIV infections, HIV infections, Cell vaccines

## Abstract

Although natural killer (NK) cells have been studied in connection with dendritic cell (DC)-based vaccination in the field of cancer immunology, their role has barely been addressed in the context of therapeutic vaccination against HIV-1. In this study, we evaluated whether a therapeutic DC-based vaccine consisting of monocyte-derived DCs electroporated with Tat, Rev and Nef encoding mRNA affects NK cell frequency, phenotype and functionality in HIV-1-infected individuals. Although the frequency of total NK cells did not change, we observed a significant increase in cytotoxic NK cells following immunisation. In addition, significant changes in the NK cell phenotype associated with migration and exhaustion were observed together with increased NK cell-mediated killing and (poly)functionality. Our results show that DC-based vaccination has profound effects on NK cells, which highlights the importance of evaluating NK cells in future clinical trials looking at DC-based immunotherapy in the context of HIV-1 infection.

## Introduction

To date, antiretroviral therapy (ART) is the only effective treatment available to reliably supress replication of human immunodeficiency virus (HIV-1) and avoid progression to acquired immunodeficiency syndrome (AIDS). However, ART cannot eliminate latently integrated virus meaning that life-long treatment is required and important disease and treatment related comorbidities persist. Discontinuation of ART results in rapid viral rebound in the majority of people living with HIV-1 (PLWH)^1^. Therefore, immune-based strategies such as therapeutic vaccination are being explored to improve the host’s immune response to control HIV-1 replication without the need of ART. Dendritic cells (DCs) are important players in this strategy as they are critical mediators of both innate and adaptive immune responses. Therefore, therapeutic vaccination with autologous monocyte-derived DCs expressing HIV-1 antigens has been explored in several clinical trials^2–4^.

Most DC-based vaccination studies focus on the analysis of T cell responses whereas natural killer (NK) cells are generally not, or only superficially studied^2,4–6^. However, NK cells play an essential role in the defence against viruses, including HIV-1, by eliminating infected cells without the need for prior sensitisation. Human NK cells are classically dichotomized into two subgroups: CD56^bright^CD16^−^ regulatory NK cells and CD56^dim^CD16^+^ cytotoxic NK cells^7^. NK cells also express a variety of germ-line encoded activating (e.g. NKG2D, NKG2C and NKp46) and inhibitory (e.g. killer immunoglobulin-like receptors [KIRs] and NKG2A) receptors, allowing them to survey the environment for expression of stress-induced ligands and self-antigens (MHC-I). NK cell functionality strongly depends on the engagement of inhibitory KIRs with MHC-I molecules in a process called NK cell education. Educated NK cells mediate enhanced cytotoxic functions upon later stimulation, whereas non-educated NK cells remain hypofunctional^8^. Interestingly, educated NK cells play a beneficial role in DC maturation and the subsequent adaptive immune response^9^. In addition, NK cells can lyse target cells through an indirect pathway using FcyRIIIa/CD16a and immunoglobulin G (mainly IgG1 and IgG3) antibodies specific for viral surface proteins via antibody dependent cellular cytotoxicity (ADCC) resulting in the release of IFN-γ, granzymes and perforins^10^. The importance of this mechanism in HIV-1 infection has been highlighted by the observation of a correlation between NK cell-mediated ADCC and delayed disease progression^11^. Furthermore, in the prophylactic RV144 vaccine trial it was shown that part of its protective effect was related to ADCC^12^.

NK cells can shape the immune response via bi-directional interactions with DCs. Thus, NK cells play an important role in the maturation of DCs by either killing immature DCs or by stimulation of DC maturation^13,14^, whereas mature DCs stimulate NK cells and induce migration of NK cells, which in turn proliferate rapidly and produce interferon (IFN)-γ^15,16^. Therefore, modulation of the NK-DC crosstalk by immunotherapy is an interesting issue to explore. Although increasing evidence reveals the importance of the NK-DC interplay in anti-HIV-1 immunity, only few DC-based vaccination studies have attempted to evaluate NK cell responses after therapeutic vaccination^17,18^. Most studies assessing NK cell responses following vaccination, focussed only on NK cell frequency, complemented by a limited analysis of NK cell phenotype prior to and after vaccination. A minority of studies also investigated the cytotoxic capacity of NK cells using MHC-devoid K562 cells as targets. However, a comprehensive functional analysis is lacking in these studies.

**Fig. 1:**
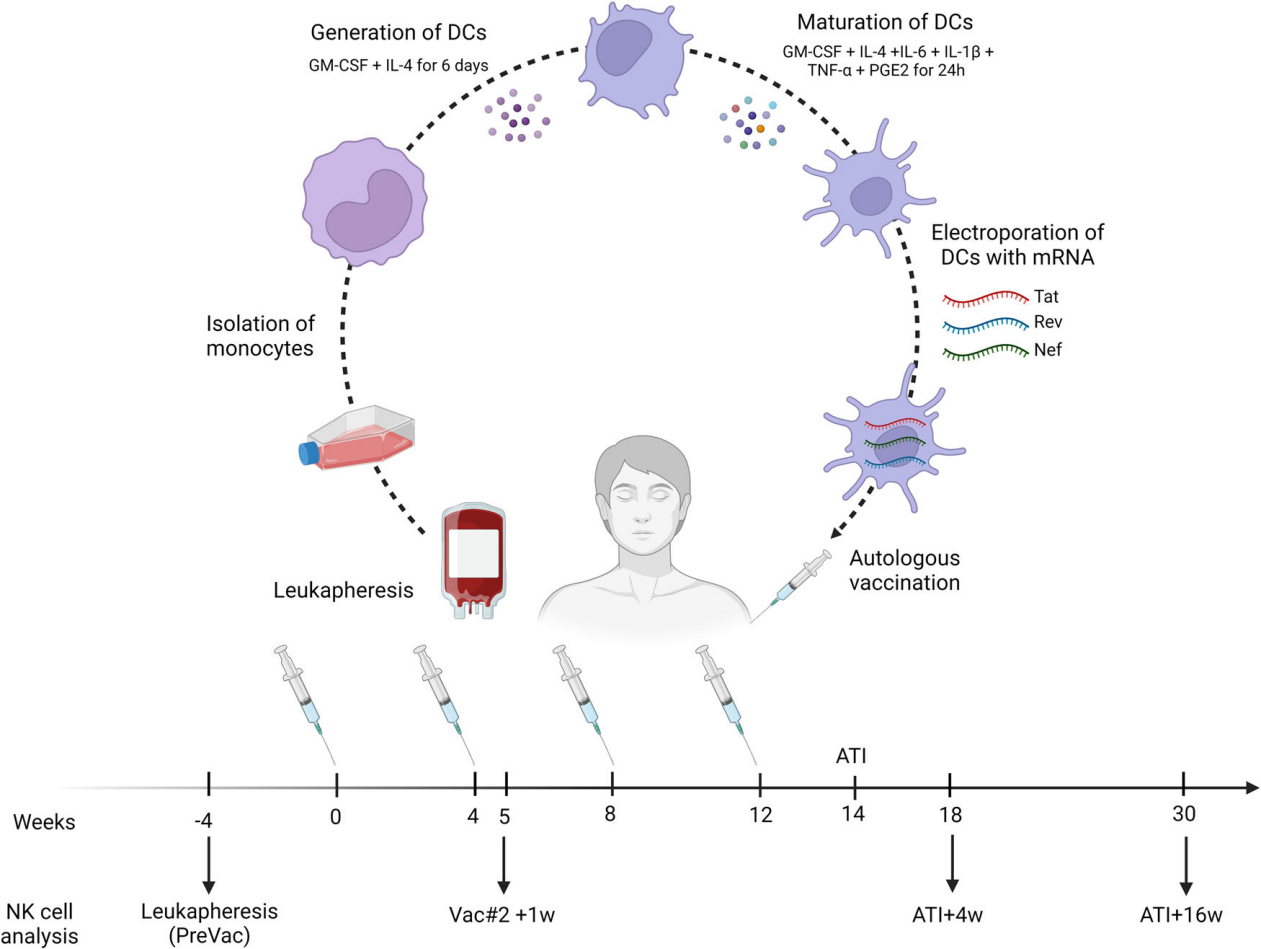
Overview of the DC-TRN study. Four weeks prior to administration of the first vaccine, a leukapheresis was performed from which monocytes were isolated and subsequently differentiated into DCs via culturing in X-vivo 15 medium supplemented with 1% heat-inactivated autologous plasma in the presence of GM-CSF and IL-4 for 6 days. On day 6, maturation of immature monocyte-derived DC was induced by the addition of a cytokine cocktail consisting of GM-CSF, IL-4, IL-6, IL-1β, TNF-α and PGE2 for 24 h. Participants received 4 identical vaccines consisting of three autologous monocyte-derived dendritic cell preparations, electroporated with either Tat, Rev or Nef mRNA with intervals of 4 weeks. The vaccines were administered both subcutaneously and intradermally at three separate places (right arm, left arm and one thigh) to exclude immunodominance of one of the antigens. Two weeks after administration of the final vaccine (week 14), participants were submitted to ATI. ART was restarted when considered necessary by the treating physician. Time points at which NK cell analysis was performed are indicated with arrows: before vaccination (PreVac), 1 week after the second vaccination (Vac#2 + 1w), 4 weeks after ATI (ATI + 4w) and 16 weeks after ATI (ATI + 16w). Figure created with BioRender.com.

In a non-randomized phase I/IIa trial, we vaccinated PLWH with autologous DCs electroporated with mRNA encoding for Tat, Rev and Nef. Next, participants were submitted to an analytical treatment interruption (ATI) (Fig. 1). Although this vaccine was shown to be safe, well-tolerated and to enhance CD4^+^ and CD8^+^ T cell responses, no significant correlation with time off treatment was observed^4^. In this study, we aimed to evaluate whether the DC-TRN vaccine affects NK cell frequency, phenotype and functionality in order to obtain new insights into the importance of the NK-DC crosstalk during therapeutic vaccination against HIV-1.

## Results

### The DC-TRN vaccine increases the frequency of cytotoxic NK cells

**Fig. 2:**
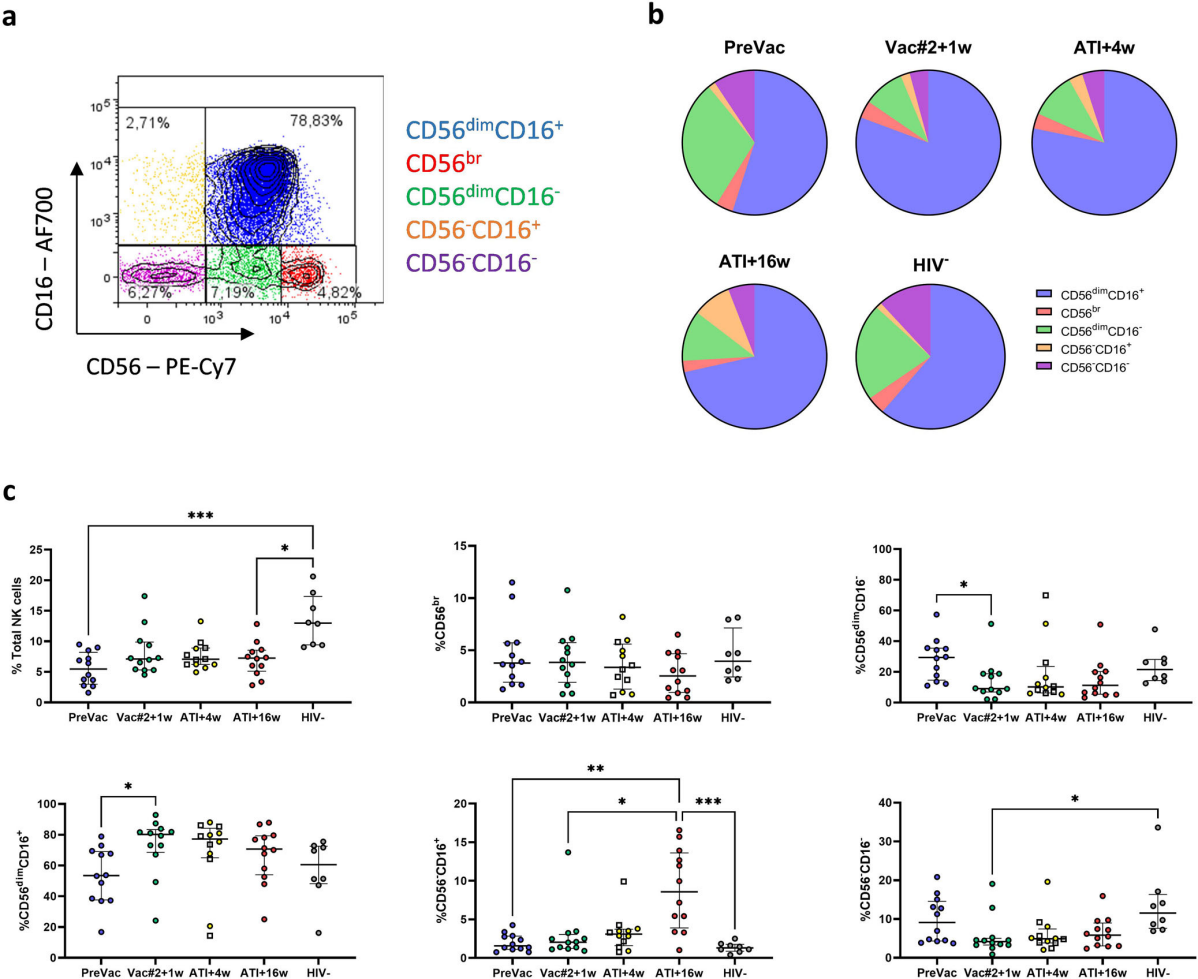
Frequency of NK cell subsets during the course of the DC-TRN trial. **a** Gating strategy used to identify different subsets of human NK cells based on CD56 and CD16 expression. **b** Pie charts showing the proportional distribution of the various NK cell subsets. **c** Frequency of total NK cells and NK cell subsets in blood during the course of the DC-TRN study (*n* = 12) and in HIV-1^−^ individuals (*n* = 8). Participants who rebounded at ATI + 4w are indicated with squares. Data are presented as median ± interquartile range (IQR), Kruskal–Wallis test with Dunn’s multiple comparisons test for post-hoc analysis. *P*-values <0.05 were considered statistically significant and graphically denoted as follows: *p < 0.05, **p < 0.005 and ***p < 0.001.

Chronic HIV-1 infection induces an abnormal distribution of NK cell subsets, with a diminished proportion of cytotoxic (CD56^dim^CD16^+^) NK cells and an expansion of dysfunctional (CD56^−^CD16^+^) NK cells^19^. We aimed to investigate the effect of DC-based vaccination on NK cell frequency and distribution since DCs are known to induce NK cell proliferation and maturation^20,21^. Based on the expression of CD56 and CD16, NK cells were subdivided into 4 subsets: CD56^br^CD16^−^, CD56^dim^CD16^−^, CD56^dim^CD16^+^ and CD56^−^CD16^+^ (Fig. 2a/Supplementary Fig. 1a). We also identified a fifth population within the CD7^+^ subset, lacking both CD56 and CD16 expression. This subset expresses some NK cell markers, including NKG2D and NKp46, but lacks KIR expression and might at least in part represent other (helper) innate lymphoid cell types^22^. (Supplementary Fig. 1b). We did not observe significant changes in the percentage of total NK cells over the different time points of the trial (‘PreVac’: before vaccination; ‘Vac2 + w1’: 1 week after the second vaccination; ‘ATI + 4w’ and ‘ATI + 16w’: 4 or 16 weeks after ATI). Nevertheless, the percentage of total NK cells was significantly lower at PreVac and ATI + 16w compared to the HIV-1^−^ cohort (Fig. 2c). A significant decrease in the CD56^dim^CD16^−^ subset together with an increase in the CD56^dim^CD16^+^ subset was observed at Vac#2 + 1w compared to PreVac. The percentage of the CD56^dim^CD16^−^ and CD56^dim^CD16^+^ subsets were not re-established to PreVac levels after ATI. The levels of CD56^br^ and CD56^−^CD16^−^ NK cells were comparable for all time points studied and were similar to those of HIV-1^−^ individuals. The dysfunctional CD56^−^CD16^+^ NK cell subset remained stable during the vaccination period and at ATI + 4w, and significantly increased at ATI + 16w, most likely due to viral rebound (Fig. 2b, c). At ATI + 4w, 6/12 (50%) of participants had rebounded (i.e. plasma viral load (pVL)>1000 copies/mL). The frequency of the studied NK cell subsets did not differ significantly between participants who either rebounded at ATI + 4w or later.

Remarkably, when we compare the NK cell frequency in participants with time to pVL rebound before and after 4 weeks of ATI, we observed that individuals with early rebound have significantly more NK cells at PreVac and ATI + 4w compared to individuals who rebounded after 6 weeks of ATI (Supplementary Fig. 2e). More specifically, these early rebounders had a higher percentage of cytotoxic NK cells and a lower percentage of cytokine producing NK cells at PreVac compared to participants who rebounded after 6 weeks (Supplementary Fig. 2a). Based on the time remaining off ART, participants were categorized as “resumers” (off ART ≤ 96 weeks) and “non-resumers” (off ART > 96 weeks). No differences in NK cell frequency between resumers and non-resumers were observed for all studied NK cell subsets and time points (Supplementary Fig. 2b, c) suggesting that NK cell frequency is not predictive of time spent off ART.

### DC-TRN vaccination alters the NK cell receptor repertoire

**Fig. 3:**
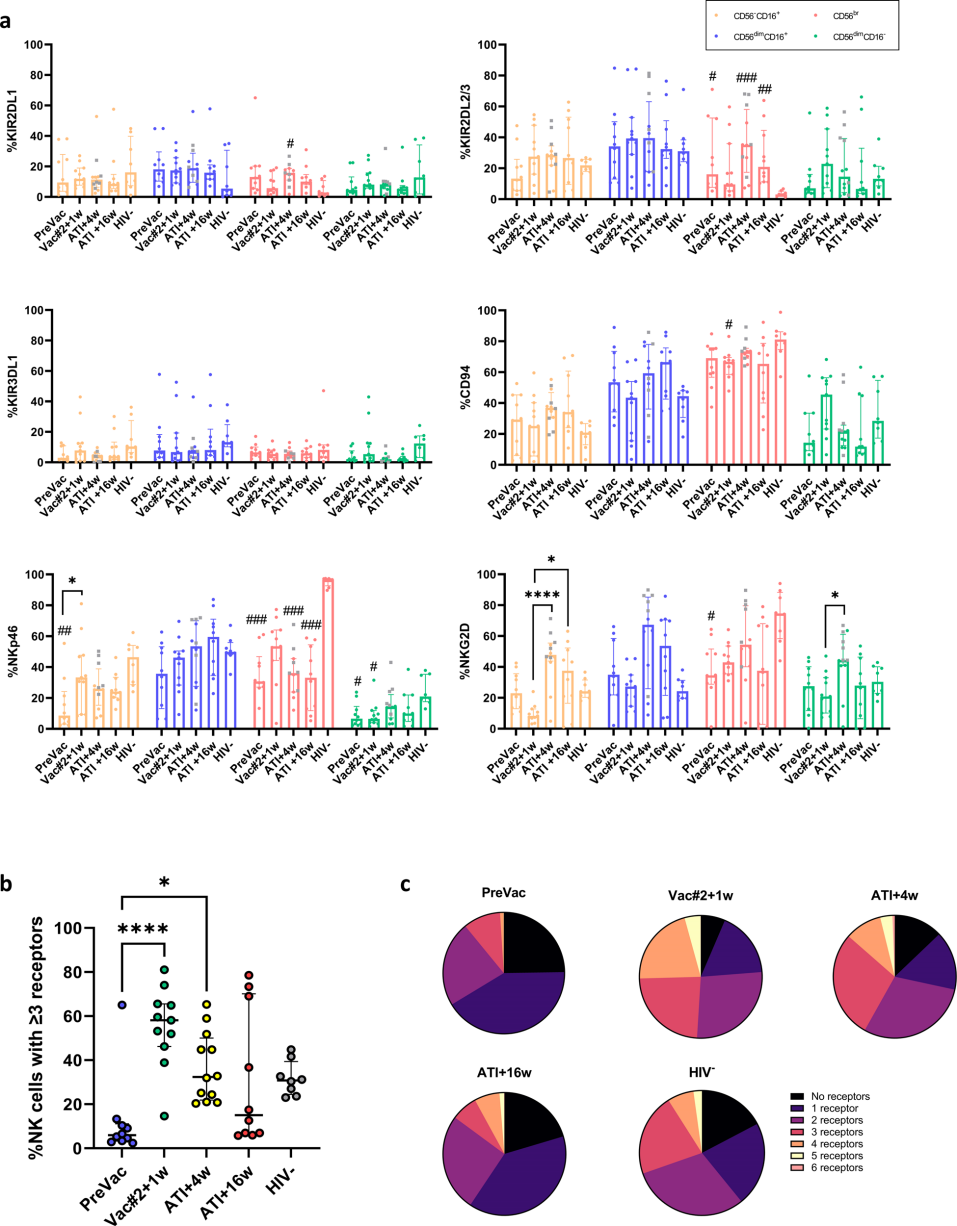
Expression of activating and inhibitory receptors on NK cells during the course of the DC-TRN trial. **a** Percentage of NK cell subsets expressing the indicated marker in participants during the trial and HIV-1^−^ individuals. Participants that rebounded at ATI + 4w are indicated with a grey square. **b** Percentage of NK cells expressing 3 or more receptors simultaneously (median ± IQR; Kruskal–Wallis test with Dunn’s multiple comparisons test for post-hoc analysis). **c** Pie charts showing the proportion of NK cells co-expressing 0–6 receptors. Significant differences between the studied time points are graphically denoted with ‘*’as follows: *p < 0.05, **p < 0.005, ***p < 0.001 and ****p < 0.0001. ‘#’ indicate a significant difference compared to the HIV-1^−^ group.

Within the previously defined NK cell subpopulations, we measured the expression of activating and inhibitory NK cell markers since these markers are linked to NK cell education, functionality and DC maturation^8,9,23^. The inhibitory KIRs, KIR2DL1, KIR2DL2/3 and KIR3DL1 were assessed as they have been well-studied in the context of protection against HIV-1^24^. Compared to PreVac, a trend towards a higher percentage of KIR-expressing CD56^dim^CD16^−^ and CD56^−^CD16^+^ NK cells and a lower percentage of KIR-expressing CD56^br^ NK cells at Vac#2 + 1w was observed. The expression of the studied KIRs at ATI + 4w between participants who experienced viral rebound before ATI + 4w was not significantly lower compared to that of participants with controlled pVL at ATI + 4w (Fig. 3a). In addition, the percentage of NK cells expressing CD94, a co-receptor for NKG2A/C receptors, remained stable during vaccination and was similar to that of HIV-1^−^ individuals for all NK cell subsets. The percentage of NKp46 expressing CD56^−^CD16^+^ NK cells significantly increased at Vac#2 + 1w, to a similar level as that of HIV-1^−^ individuals and remained elevated after ATI. Within the same subset of NK cells, participants who had rebounded by ATI + 4w showed a higher percentage of NKp46^+^ NK cells compared to participants with longer suppression of pVL. Moreover, overall levels of NKG2D^+^ NK cells were higher at ATI + 4w compared to PreVac for all subsets. At ATI + 4w, the percentage of NK cells expressing NKG2D was significantly higher in participants who had experienced viral rebound compared to those who did not. Furthermore, the percentage of NK cells co-expressing 3 or more receptors was significantly higher at Vac#2 + 1w and ATI + 4w compared to PreVac and resembles the profile of HIV-1^−^ individuals. However, this percentage decreased again at ATI + 16w (Fig. 3b, c and Supplementary Fig. 3).

### The DC-TRN vaccine induces a less exhausted and enhanced migratory profile in NK cells

**Fig. 4:**
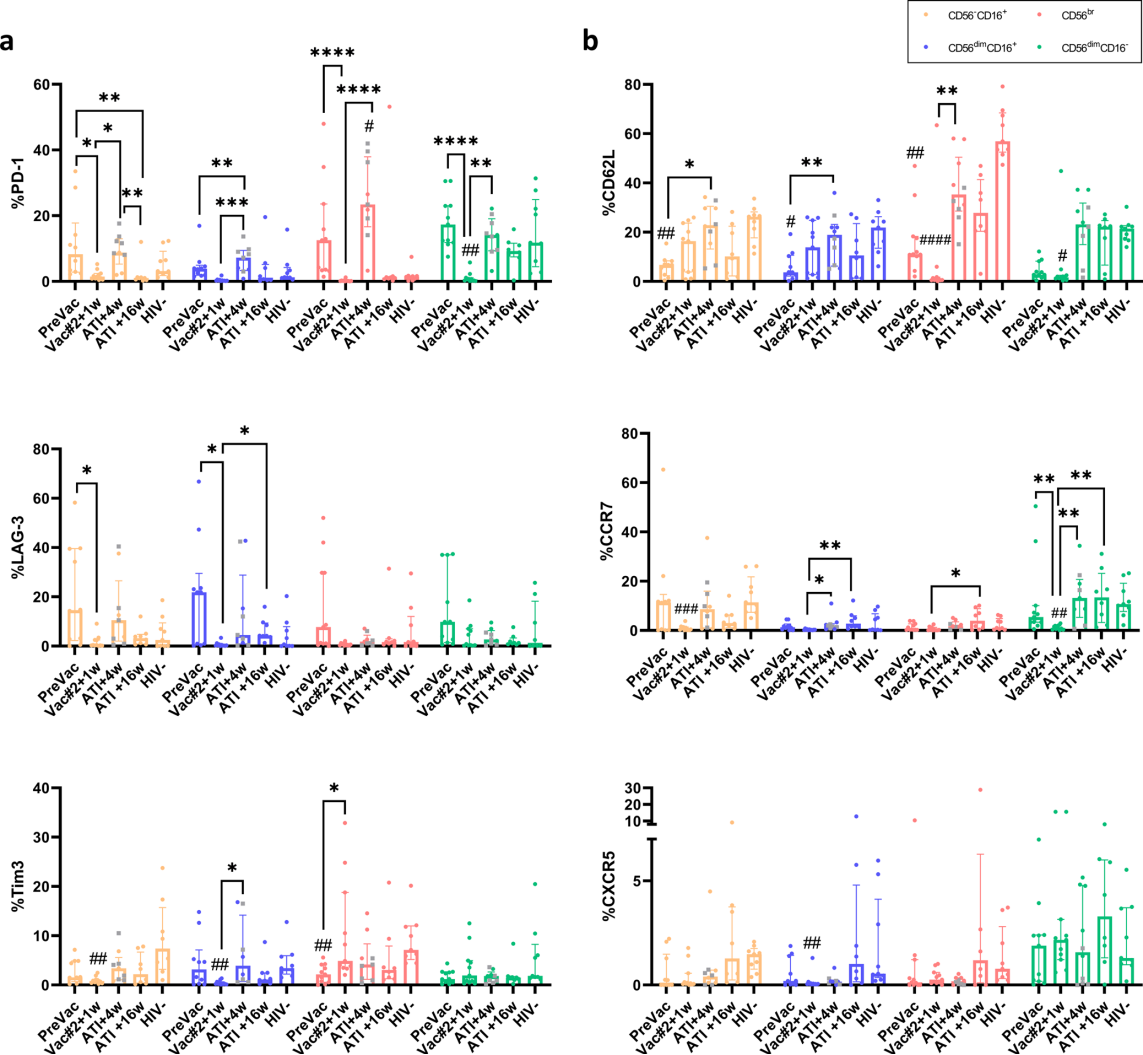
Migratory and exhaustion profile of NK cells during the course of the DC-TRN trial. Percentage of NK cell subets expressing the markers involved in (**a**) exhaustion and (**b**) homing to LN follicles in participants during the trial and in HIV-1^−^ individuals. Median ± IQR; Kruskal–Wallis test with Dunn’s multiple comparisons test to compare the studied time points and HIV-1^−^ individuals within the different NK cell subsets. Participants that rebounded at ATI + 4w are indicated with a grey square. Comparison between participants that rebounded at ATI + 4w or not was performed using Mann–Whitney *U*-test. Significant differences between the studied time points are graphically annotated with ‘*’as follows: *p < 0.05, **p < 0.005, ***p < 0.001 and ****p < 0.0001. ‘#’ indicate a significant difference compared to HIV-1^−^ group.

Besides KIRs, NK cells also express immune checkpoint molecules (ICMs) which are important mediators of immune tolerance^25^. During chronic infections, increased signalling through ICMs occurs which ultimately leads to an exhausted state of effector immune cells^26^. While this phenomenon has been extensively studied for T cells, the concept of exhaustion has not been widely described for NK cells^27^. In the current study, the percentage of NK cells expressing programmed cell death 1 (PD-1), lymphocyte activation gene 3 (LAG-3) and T cell immunoglobulin and mucin-domain containing 3 (Tim3) followed a similar trend for all NK cell subsets. First, a decrease was observed at Vac#2 + 1w compared to PreVac after which a renewed increase at ATI + 4w was observed (Fig. 4a). Interestingly, participants who rebounded at ATI + 4w showed a significantly higher expression of PD-1 in comparison with participants with controlled pVL. For LAG-3 and Tim3 no significant difference was observed between participants with pVL rebound at ATI + 4w or not. Remarkably, all ICMs declined again at ATI + 16w, although all participants had experienced a viral rebound by that time (Fig. 4a).

In addition, the capacity of NK cells to migrate towards the lymph node (LN) follicles was assessed as this is one of the important viral reservoir sites^28^. The percentage of NK cells expressing CD62L, a marker for LN migration, increased after ATI + 4w compared to PreVac in all NK cell subsets and decreased again at ATI + 16w. As expected, the expression of CCR7 and CXCR5 in the periphery was lower than that of CD62L. Both CCR7 and CXCR5 expression increased after ATI + 4w or ATI + 16w in all NK cell subsets (Fig. 4b). No significant differences were observed between participants who rebounded at ATI + 4w or later.

### CD30 expression is not predictive for viral rebound

Based on the observations made by Henrich et al., showing that CD30 expression on both T cells and NK cells is predictive for viral rebound in PLWH with early ART initiation, we decided to examine CD30 expression on NK cells in our study group^29^. Our results show limited expression of CD30 on all NK cell subsets analysed at the different time points, which was similar to that observed for HIV-1^−^ individuals. No significant difference was observed between participants with suppressed viremia and viral rebound at ATI + 4w (Supplementary Fig. 4).

### No increase in the frequency of memory-like NK cells upon DC-TRN vaccination

**Fig. 5:**
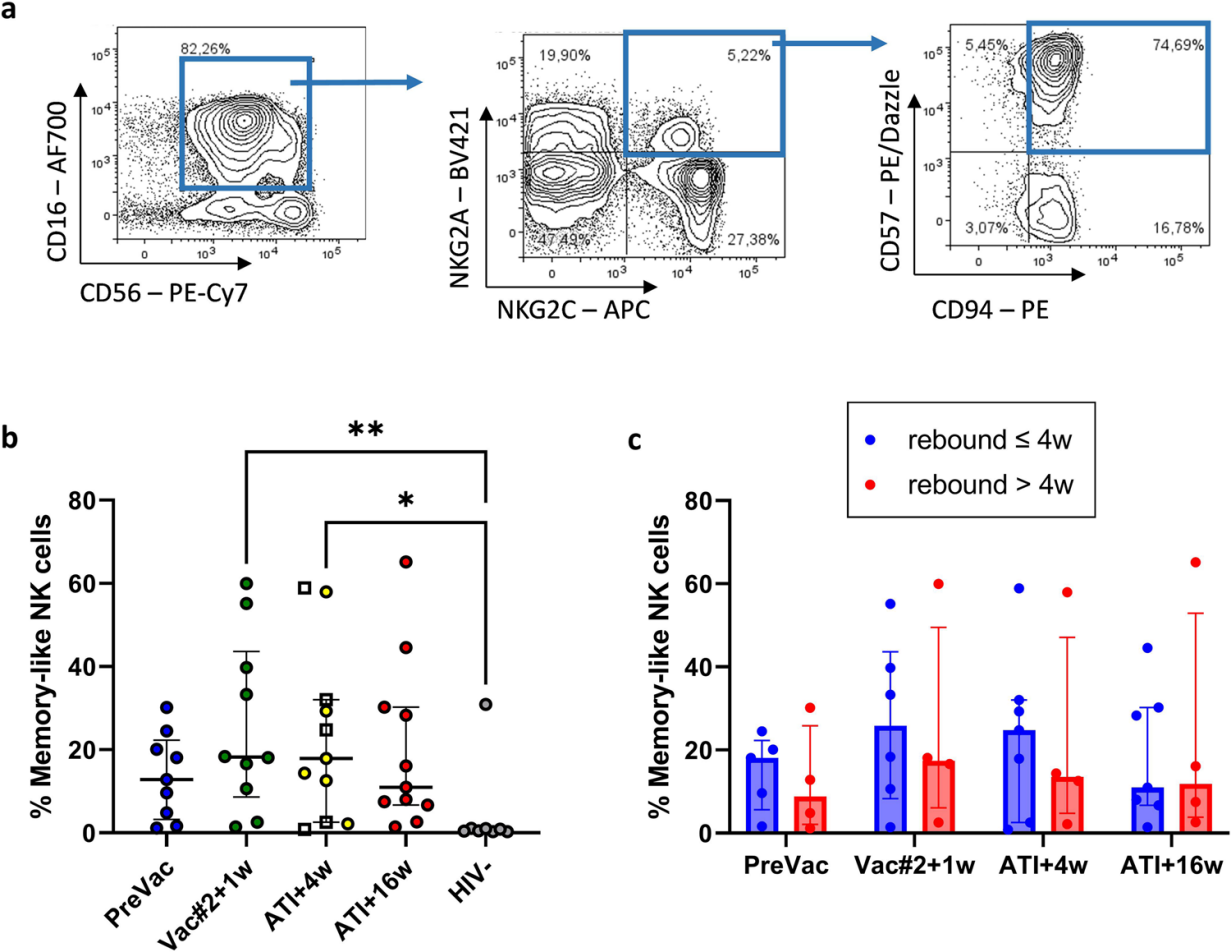
Frequency of memory-like NK cells. **a** Gating strategy on a representative participant for determining memory-like NK cells. Gating was performed on viable CD56^dim^CD16^+^ NK cells and subsequently on NKG2A^−^, NKG2C^+^, CD94^+^ and CD57^+^ NK cells. **b** Frequency of memory-like NK cells in blood of DC-TRN participants during the course of the trial compared to uninfected individuals (HIV-1^−^). Median ± IQR; Kruskal–Wallis test with Dunn’s multiple comparison test. Squares indicate participants who have rebounded at ATI + 4w. **c** Comparison in percentage of memory-like NK cells between participants who rebounded before or after 4 weeks of ATI. *P*-values <0.05 were considered statistically significant and graphically annotated as follows: *p < 0.05 and **p < 0.005.

Recent data show that NK cells have certain adaptive features and can develop memory-like properties^30,31^. However, the effect of DC-vaccination on memory NK cells has not been studied so far. Therefore, we investigated whether our vaccine could increase the proportion of memory-like NK cells. Memory-like NK cells were identified as CD56^dim^CD16^+^, NKG2C^+^, NKG2A^−^, CD57^+^ and CD94^+^ (Fig. 5a). Our results show that vaccination did not significantly alter the frequency of memory-like NK cells. However, compared to PreVac, we observed a trend towards an increase in memory-like NK cells at Vac#2 + 1w and ATI + 4w. Moreover, the percentage of memory-like NK cells at Vac#2 + 1w and ATI + 4w was significantly higher compared to HIV-1^−^ individuals. No significant difference in memory-like NK cells was detected between participants that had rebounded (median of 25%) or not (median of 16%) at ATI + 4w (Fig. 5b). Additionally, the percentage of memory-like NK cells in participants who rebounded before or after 4 weeks of ATI did not differ significantly. However, a trend towards a higher frequency of memory-like NK cells in participants experiencing an early rebound was observed at PreVac, Vac#2 + 1w and ATI + 4w (Fig. 5c). Similar observations were made for resumers and non-resumers (Supplementary Fig. 5d).

When evaluating the expression of the individual markers, we observed that the percentage of NK cells expressing NKG2C and NKG2A remained stable during vaccination and after ATI. Compared to PreVac, a significantly reduced expression of the NK cell maturation marker CD57 was observed for the CD56^br^ subset at Vac#2 + 1w which subsequently returned to baseline levels at ATI + 4w and ATI + 16w. In comparison, HIV-1^−^ individuals had significantly lower frequencies of CD57^+^ CD56^br^ NK cells (Supplementary Fig. 5a–c).

### Direct NK cell-mediated cytotoxicity is not affected by DC-based vaccination

**Fig. 6:**
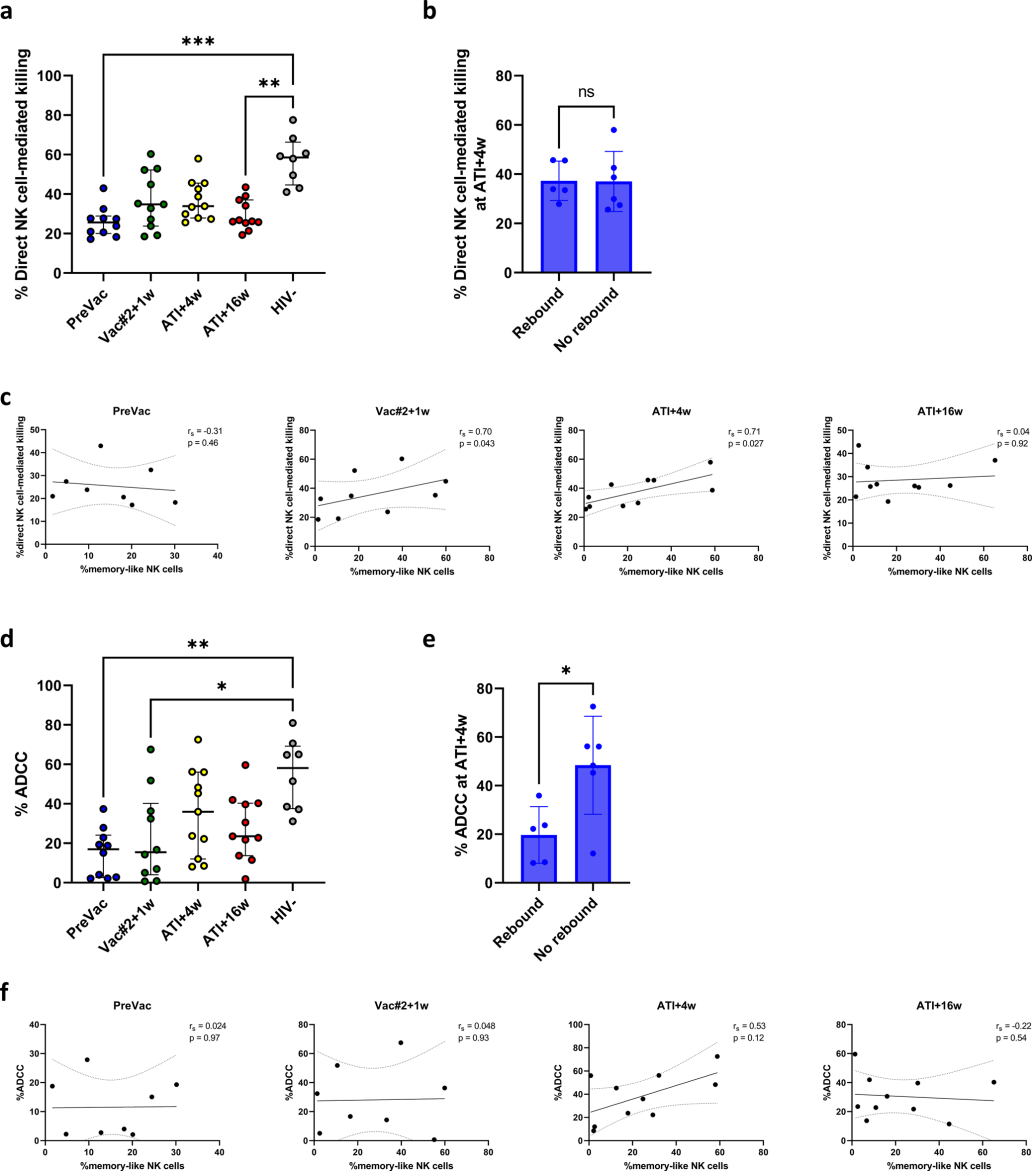
Direct and indirect NK cell-mediated cytotoxicity upon DC-TRN vaccination. **a** PBMCs from trial participants or HIV-1^−^ individuals were incubated with K562 cells (in 10:1 effector:target ratio) for 6 h. Viability of K562 cells was assessed using flow cytometry (FVS780 staining). Median ± IQR; Kruskal–Wallis with Dunn’s multiple comparisons. **b** Comparison of cell death at ATI + 4w between participants who rebounded or not (median ± IQR; Mann–Whitney *U*-test). **c** Correlation between frequency of memory-like NK cells and direct NK cell-mediated killing of K562 cells for the different time point of the trial (Spearman correlation coefficients and *p*-values (two-sided) are indicated within the graphs. Dashed lines indicate 95% confidence interval). **d** PBMCs from trial participants or uninfected individuals (HIV-1^−^) were incubated with CEM.NKr CCR5^+^ cells coated with recombinant gp120 in the presence of 1 µg/mL VRC01 for 12 h. Cell death was measured using flow cytometry (FVS780) and %ADCC was calculated. Median with IQR; Kruskal–Wallis test with Dunn’s multiple comparisons test. **e** Comparison of %ADCC at ATI + 4w between participants who rebounded at this point or not (median ± IQR; Mann–Whitney *U*-test). **f** Correlation between frequency of memory-like NK cells and ADCC-mediated killing for the different time point of the trial (Spearman correlation coefficients and *p*-values (two-sided) are indicated within the graphs. Dashed lines indicate 95% confidence interval). *P*-values <0.05 were considered statistically significant and graphically annotated as follows: *p < 0.05, **p < 0.005 and ***p < 0.001.

The direct killing capacity of NK cells was assessed using K562 cells as target cells in a 6-h co-culture with PBMCs (Supplementary Fig. 6a). The median direct killing capacity of NK cells in a 10:1 E:T ratio was 35% at Vac#2 + 1 and ATI + 4w, which was higher compared to PreVac (26%), although not reaching statistical significance. As expected, cell death levels declined again at ATI + 16w, which could be related to viral rebound (Fig. 6a and Supplementary Fig. 7a). The same trend was observed for the 3:1 ratio (Supplementary Fig. 6b). For both E:T ratios, the killing capacity of NK cells from HIV-1^−^ donors was significantly higher than that of PreVac and at ATI + 16w for the 10:1 ratio. At ATI + 4w, we did not find a significant difference in cell death between participants that had rebounded by ATI + 4w or later. Moreover, no difference in cell death was observed when comparing resumers versus non-resumers (Supplementary Fig. 6c, d). Interestingly, a significant positive correlation was observed between NK cell-mediated killing of K562 cells and the frequency of memory-like NK cells at Vac#2 + 1w and ATI + 4w but not at PreVac and ATI + 16w (Fig. 6c). Furthermore, a trend towards a positive correlation between NK cell-mediated killing of K562 cells and the frequency of CD56^dim^CD16^−^ NK cells was observed. In contrast, a negative correlation with CD56^dim^CD16^+^ NK cell frequency was observed for all time points (Supplementary Fig. 7b).

### Enhanced ADCC-mediated cytotoxicity and polyfunctionality upon vaccination

We first studied ADCC-mediated cytotoxicity using a well-established system consisting of a B cell lymphoma cell line (Raji cells) co-cultured with PBMCs in the presence of the anti-CD20 monoclonal antibody rituximab. Here, a significant increase in ADCC-mediated killing was observed at Vac#2 + 1w compared to Prevac, which decreased at ATI + 4w and ATI + 16w (Supplementary Fig. 8a). Participants that experienced viral rebound before ATI + 4w had lower ADCC-mediated killing capacity compared to participants with no viral rebound by that time (Supplementary Fig. 8b, c).

Next, we assessed the ADCC-mediated killing capacity of the participant’s NK cells using a model in which PBMCs were co-cultured with CEM.NKr CCR5^+^ cells coated with recombinant gp120_BaL_ in the presence of the broadly neutralizing VRC01 antibody. We observed significantly lower ADCC-mediated killing of CEM.NKr CCR5^+^ cells at PreVac and at Vac#2 + 1w compared to uninfected donors. The ADCC capacity was restored at ATI + 4w and was no longer significantly different from the HIV-1^−^ cohort (Fig. 6d). Interestingly, ADCC was significantly lower in participants who rebounded at ATI + 4w compared to those who did not rebound (Fig. 6e). There was a trend towards a higher ADCC capacity of NK cells in participants who rebounded after 4 weeks compared to those who rebounded before (Supplementary Fig. 9a, b). However, no differences in ADCC-mediated cytotoxicity of either Raji or CEM.NKr CCR5^+^ cells could be observed between resumers and non-resumers for all studied time points, indicating that increased ADCC is not predictive of time off ART (Supplementary Figs. 8d and 9c). Moreover, ADCC-mediated killing increased with increasing frequency of CD56^dim^CD16^+^ NK cells (Supplementary Fig. 9d). For all time points evaluated, a correlation between direct and ADCC-mediated NK cell killing was not observed (Supplementary Fig. 12a). A trend towards a positive correlation between ADCC-mediated killing and the frequency of memory-like NK cells was found at ATI + 4w (Fig. 6f).

**Fig. 7:**
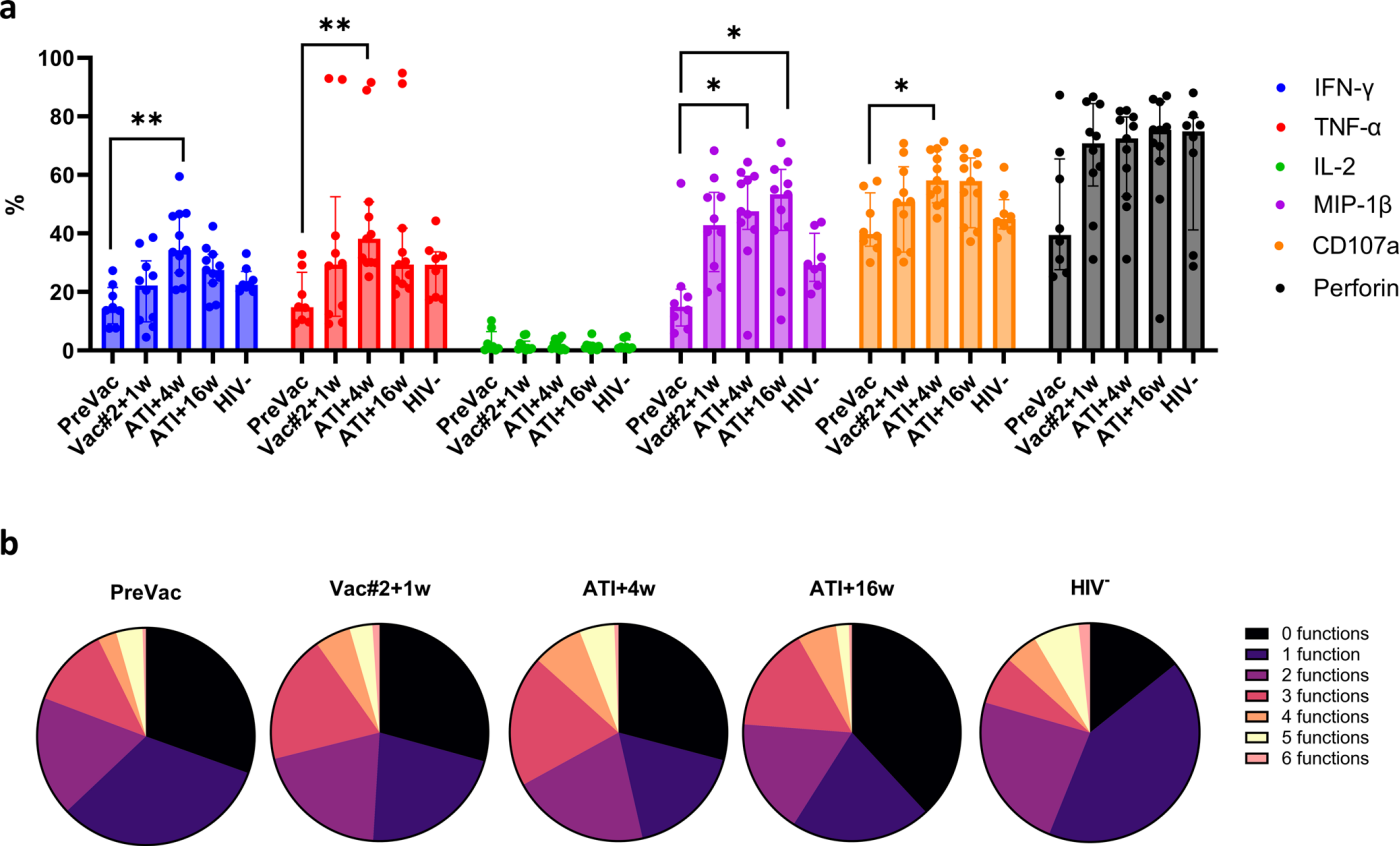
ADCC-induced NK cell polyfunctionality upon DC-TRN vaccination. PBMCs from trial participants or uninfected individuals (HIV-1^−^) were incubated with CEM.NKr CCR5^+^ cells coated with recombinant gp120 in the presence of 1 µg/mL VRC01 for 12 h. NK cells were identified as described in Supplementary Fig. 1a and subsequent gating for intracellular markers was performed (see Supplementary Fig. 10). **a** Percentage of NK cells expressing the individual intracellular markers in trial participants and HIV-1^−^ individuals. Data are presented as median ± IQR; Kruskal–Wallis with Dunn’s multiple comparisons test for each marker separately. **b** Pie charts showing the proportion of NK cells having 0–6 functions. *P*-values <0.05 were considered statistically significant and graphically annotated as follows: *p < 0.05 and **p < 0.005.

Polyfunctionality has become a well-established concept to assess the quality of T cell responses in PLWH^32^. For NK cells, this concept has also been described in a limited number of studies^33,34^. We therefore assessed the (poly)functionality of NK cells using ICS for CD107a, TNF-α, IFN-γ, IL-2, MIP-1β and perforin (Supplementary Fig. 10a, b). Except for IL-2, a trend towards increased levels of the studied markers was observed at Vac#2 + 1w compared to PreVac. This percentage further increased at ATI + 4w where it was significantly higher compared to PreVac for CD107a, TNF-α, IFN-γ and MIP-1β (Fig. 7a). No significant differences in the percentage of NK cells with 0–3 functions were observed between PreVac, Vac#2 + 1w and both time points after ATI, whereas a significant increase in the percentage of NK cells with 4 and 5 functions was observed at ATI + 4w compared to PreVac. This increase was mainly attributed to the contribution of TNF-α, IFN-γ, perforin and CD107a. (Fig. 7b and Supplementary Fig. 11a, b). At ATI + 4w, participants who remained virally suppressed showed a significantly higher percentage of NK cells with 5–6 functions compared to participants who displayed viral rebound (Supplementary Fig. 11a). We identified a significant positive correlation between ADCC-mediated killing and the percentage of polyfunctional NK cells (defined as ≥4 functions) at Vac#2 + 1w and ATI + 4w and a negative correlation at PreVac and ATI + 16w (Supplementary Fig. 12b).

Whereas T cells express both CD8α and β, a subpopulation of NK cells only express the CD8α homodimer^35^. CD8α has been reported as a marker for NK cell polyfunctionality^33^. We observed a significantly increased CD8α expression on CD56^dim^CD16^−^ NK cells after ATI + 4w and ATI + 16w. This was however not observed for other NK cell subsets (Supplementary Fig. 11c). No significant differences in CD8α expression between participants and HIV-1^−^ individuals were identified. Moreover, we did not find a positive correlation between CD8α expression and polyfunctionality (Supplementary Fig. 12c).

### Increased levels of KIR2DL1^+^ NK cells result in enhanced NK cell cytotoxicity after the second vaccination

KIRs represent important regulators of NK cell activity via interactions with HLA class I molecules. The ligands of KIR2DL1, KIR2DL2/3 and KIR3DL1 are HLA-C2, HLA-C1 and HLA-B, respectively^36^. HLA-A/B/C genotyping showed that all participants have at least two HLA-KIR matches except for study participant H014, who only has one matching combination (Supplementary Table 1). To investigate whether and how NK cell functionality is affected by changes in KIR expression, we subdivided the participants based on an increased or decreased percentage of KIR^+^ NK cells compared to PreVac. There were no significant changes in direct NK cell-mediated killing between participants with increased or decreased expression of KIRs. However, ADCC-mediated cytotoxicity was increased in participants with increased KIR2DL1 frequency between PreVac, ATI + 4w and ATI + 16w. This increase was most outspoken in participants with education-competent KIR-HLA combinations, suggesting that licensing through KIR2DL1 results in increased functionality of NK cells (Supplementary Figs. 13, 14).

## Discussion

In this study, we investigated whether autologous DC-based immunotherapy affects NK cell frequency, phenotype and functionality. Although we did not observe a difference in the frequency of total NK cells, we found significant redistributions of NK cell subsets upon DC-based vaccination and subsequent ATI. At Vac#2 + 1w, we identified an increase in the cytotoxic NK cell subset concomitant with a decrease in the CD56^dim^CD16^−^ subset. The decrease in CD56^dim^CD16^−^ NK cells in blood might be explained by the migration of NK cells to the LN where interaction with DCs is likely taking place. Another possible explanation would be the maturation of CD56^dim^CD16^−^ NK cells into CD56^dim^CD16^+^ NK cells^37^. The latter possibility is less likely since a trend towards reduced CD57 expression was observed for all NK cell subsets at Vac#2 + 1w compared to baseline. Di Nicola et al. also reported a significant increase in cytotoxic NK cells upon vaccination against B cell lymphoma using autologous tumour-loaded DC^21^. However, this is in contrast with the findings of Sarhan et al. that DC-based vaccination against melanoma results in a significant decrease in CD16 expression on NK cells^38^. These discordant results could be attributed to differences in the protocols for DC maturation. In addition, internalization or shedding of CD16 upon interaction with IgG is a well-described mechanism to overcome excessive immune stimulation^39^. Several studies reported a loss of CD56^br^ NK cells and an increase in the dysfunctional CD56^−^ population when PLWH are experiencing high pVL, which is in line with our results^19,40,41^.

Although the frequency of cytotoxic NK cells significantly increased at Vac#2 + 1w, their expression of both activating and inhibitory receptors did not change. In contrast, based on KIR expression, the CD56^dim^CD16^−^ subset showed a more educated phenotype. Kristensen et al. showed that educated NK cells are superior in terms of their capacity to mediate cytotoxic functions upon direct and anti-HIV-1 antibody dependent stimulation^23^. Our results showed a trend towards increased direct and indirect killing of target cells at Vac#2 + 1w. However, this effect did not persist after ATI, which is in line with the observation that PLWH with high viremia show decreased cytotoxicity via both direct and indirect pathways^40^.

Although in a previous study, we observed vaccine-induced antigen-specific CD8^+^ T cell responses^4^ and in the current study, profound changes in phenotype and functionality of NK cells upon vaccination were observed, no prolonged time off ART following ATI was observed compared to historical controls. Importantly, the trial was performed in 2007 meaning that guidelines to restart ART following ATI were less strict compared to current criteria, allowing longer periods off ART (28–352 weeks) with increased pVL making our samples unique^4^. The timing to restart ART was based on the judgement of the treating physician, which could introduce a level of bias. Nevertheless, NK cell frequency, phenotype and functionality did not seem to be predictive for time to viral rebound. In addition, the available antiviral drugs evolved over time, especially the introduction of integrase-inhibitors over a decade ago. Another important limitation of this study is the lack of a time point after the fourth vaccination where all participants are still virally suppressed.

T cell polyfunctionality is a well-established concept within the field of cancer and HIV-1 immunology. However, for NK cells, this concept has not been extensively studied and the few studies that have addressed “polyfunctionality” generally limit their analysis to a restricted number of markers. Thus, we are the first to perform a bona fide polyfunctionality analysis on NK cells, based on the extended analysis of T cell polyfunctionality published by Hersperger et al*.*^42^. We found that NK cells with enhanced functionality (4–5 functions) at Vac#2 + 1w were significantly increased in number. T cell polyfunctionality has been shown to correlate with better control of viral replication and slower disease progression^32^. Interestingly, Ahmad et al. showed that CD8^+^ NK cells exhibit higher polyfunctionality which was associated with a slower disease progression in untreated PLWH^33^. At Vac#2 + 1w and ATI + 16w, we found a trend towards a positive correlation between frequency and polyfunctionality of CD8^+^ cytotoxic NK cells which was absent at baseline.

In order to eliminate HIV-1-infected cells, NK cells need to be functional. ICMs are an important class of receptors hampering this functionality upon interaction with the respective ligand. In contrast to CTLs, in NK cells, the expression of ICM and the phenomenon of exhaustion have not been studied in great detail, neither in the context of cancer nor in PLWH. Norris et al. reported that the frequency of PD-1^+^ NK cells increases during HIV-1 infection and is only partially restored upon ART initiation resulting in reduced proliferative and functional capacity^27^. At Vac#2 + 1w, we observed a significant decrease in ICM which did not persist after ATI. Immune checkpoint inhibitors are rapidly becoming the treatment of choice for several cancer types, whereas only few clinical studies have been performed in the context of HIV-1. Recently, it was shown that anti-PD-1 treatment also has latency reversal effects^43^. Combining latency reversal with DC-based vaccination is an interesting approach worth exploring in the context of HIV-1 cure. In this regard, Gay et al. recently showed that Vorinostat combined with an autologous DC-based vaccine resulted in increased T cell responses and a reduction in viral reservoir size^44^. However, NK cell responses were not investigated in this study.

NK cells are normally excluded from LN follicles (LNF), so in order to control the viral reservoir, NK cells need to migrate towards LNF. Expression of CD62L facilitates homing to secondary lymphoid organs by initiating the tethering and rolling on the endothelium. Martin-Fontecha et al. showed that injection of mature DCs induced migration of NK cells to LNF in a CD62L-dependent manner^45^. Although CD62L has been suggested to be expressed mainly on CD56^dim^ NK cells, we found the highest expression on the CD56^br^ subset. While the percentage of CD62L and CCR7 increased on cytotoxic NK cells at Vac#2 + 1w, CXCR5 expression was not altered. This could be attributed to the fact that we studied NK cells in PBMCs instead of lymphoid NK cells. It has been shown that a significant number of blood NK cells in African Green monkeys is strongly positive for the LNF homing marker CXCR5, leading to control of the viral reservoir^46^. Similar data were recently obtained in a model for chronic SHIV infection. Moreover, in human samples, significantly increased numbers of CXCR5^+^ NK cells were found in LNF from PLWH compared to healthy donors^47,48^. The size of the viral reservoir was, however, not determined in this study.

Immunological memory has traditionally been known to be an “exclusive” hallmark of adaptive T and B cells. However, this paradigm has been challenged by increasing evidence indicating that NK cells possess certain features of adaptive immunity as well. Different groups have identified adaptive NK cells in mouse models for hapten-induced hypersensitivity and viral infections (MCMV, HIV-1 and Influenza)^49–51^. In a primate model of SIV, these adaptive NK cells were shown to lyse Gag- and Env-pulsed autologous DCs up to 5 years after vaccination^31^. In humans and primates, NKG2C is thought to be involved in antigen specificity (presented by HLA-E)^52^. However, this needs to be further clarified. Our results showed no significant increase in memory-like NK cells, but a trend towards a positive correlation with direct and indirect cytotoxicity was observed. Importantly, the CMV status of participants should be taken into account as this might trigger memory-like NK cell responses^53^. Unfortunately, this information was not available for the majority of our study participants. Moreover, peripheral blood might not be an ideal site to study adaptive NK cells as these cells have been shown to reside in the liver and spleen in SIV models^31^.

The administration route and mode of vaccine-mediated antigen presentation are critical for DC stimulation and subsequent crosstalk with NK cells. In the present study, an ex vivo loaded autologous DC vaccine was administered both subcutaneously and intradermally, whereas peptide-loaded DCs administered via subcutaneous injection is believed to induce the best NK cell responses in mice^45,54^. In addition, DCs that have been pulsed with the HIV-1 accessory protein Nef have previously been shown to increase IFN-γ production by CD56^br^ NK cells as well as reduce cytotoxicity of CD56^dim^CD16^+^ NK cells in vitro^55^.

Ex vivo loading of autologous DCs with HIV-1 antigens is quite costly and requires extensive expertise and infrastructure. This has led to the development of nanoparticle-based mRNA vaccines, which have been proven to be very effective in the context of SARS-CoV-2^56^. Moreover, an increase in NK cell frequency, with increased IFN-γ production was observed following BNT162b2 vaccination, which suggests it is worth investigating these nanoparticle-based mRNA vaccines against HIV-1 as well^57^.

In conclusion, our results demonstrate that DC-based immunotherapy resulted in an increased frequency of cytotoxic NK cells. Moreover, NK cells had a more licensed and migratory profile which translated into increased cytotoxicity and polyfunctionality. Since no delay in time to viral rebound was observed, these results warrant further research to optimize the efficacy of vaccine-induced NK cell responses. Overall, this work highlights the importance of in-depth analysis of NK cell frequency, phenotype and functionality after and/or during DC-based immunotherapy, which has an impact on future HIV-1 cure research. Currently, the main focus of DC-based immunotherapy is on the improvement of adaptive immune responses. Our results indicate that it is important to extend analysis to NK cell-mediated responses, and perhaps to modify DCs in such a way that these responses are more tailored towards enhanced NK cell responses. This is not only of great importance in the field of infectious diseases but can be easily expanded to other fields, such as cancer immunotherapy, as well.

## Methods

### Patient samples and study design

Between 2006–2007, study participants were recruited at Universitair Ziekenhuis Brussel (UZB) and Erasmus Medical Center (EMC) Rotterdam and provided written informed consent. The study was approved by the institutional boards of UZ Brussel (VUB 05–001) and EMC (METC 2005–227). The non-randomized Phase I/IIa trial was conducted in full conformity with the principles expressed in the Declaration of Helsinki and registered at the Netherlands Trial Register (NTR2198). Detailed trial protocol and characteristics of study participants have been described in Allard et al*.*^4^. Briefly, seventeen HIV-1 (subtype B) infected individuals with undetectable pVL were vaccinated with the DC-TRN vaccine on 4 occasions (both subcutaneously (50% of the volume) and intradermally (50% of the volume)) and 4 weeks apart (Fig. 1). ATI was initiated 2 weeks after the last vaccine administration. ART was restarted when the CD4^+^ T cell count decreased below 50% of the baseline value or after decision of the treating physician according to the guidelines for the use of ART in PLWH. Rebounders were defined as people with a detectable pVL (>50 copies/ml). Resumers were defined as people that re-initiated ART by week 96, the end of the study as defined in the protocol. This is however arbitrary, as ART resumption could be the result of various independent factors (CD4^+^ T cell count, pVL, doctor’s decision or patient’s request).

Blood was collected at several time points during the trial and PBMCs were isolated via Ficoll density gradient centrifugation. Isolated PBMCs were stored in liquid nitrogen until further use. The remaining material from the DCTRN trial, was stored in a registered biobank which was approved by the Erasmus MC Medical Ethical Committee (MEC-2022–0060). Due to limited viability and availability of some samples, only 12 of the 17 participants were assessed in the current study. Moreover, some time points are missing for some of the participants. Uninfected (HIV-1^−^) healthy donors (*n* = 8) were recruited at UZB and provided written informed consent prior to blood donation (2019/247). HIV-1^−^ donors were not matched for age and gender. Patient demographics are shown in Supplementary Table 2.

### Cell lines

K562 cells and Raji cells (kindly provided by Prof. K. Breckpot, Vrije Universiteit Brussel, Brussels, Belgium) and CEM.NKr CCR5^+^ cells (kindly provided by Prof. C. Devaux, Luxembourg Institute of Health, Luxembourg) were cultured in Roswell Park Memorial Institute (RPMI) 1640 (VWR, Leuven, Belgium), supplemented with 10% Fetal Bovine Serum (FBS, TicoEurope, Amstelveen, the Netherlands), 2 mM L-glutamine (Sigma-Aldrich, Ghent, Belgium), 100 µg/mL Streptomycin (Sigma-Aldrich) and 100 U/mL Penicillin (Sigma-Aldrich) in a humified incubator at 37 °C and 5% CO_2_.

#### HLA genotyping

Genomic DNA was extracted from PBMCs using the QiAamp DNA blood mini kit (Qiagen Benelux, Venlo, the Netherlands) according to the manufacturer’s protocol. High-resolution sequencing spanning exons 1 to 5 from HLA-A, -B and -C antigens was performed. Both the polymerase chain reaction (PCR) amplification and sequencing reagents were purchased from Applied Biosystems (Gent, Belgium)^58^.

### Direct NK cell-mediated killing assay

PBMCs from trial participants were thawed and rested overnight in Iscove’s Modified Dulbecco’s medium (IMDM, Lonza, Basel, Switzerland) containing 10% FBS, 2 mM L-glutamine, 100 U/mL Penicillin, 100 µg/mL streptomycin and 20 U/mL recombinant human IL-2 (Proleukin, Clinigen, Trent, United Kingdom), further referred to as complete IMDM, at a density of 2 × 10^6^ cells/mL in a 37 °C humidified 5% CO_2_ incubator. Co-cultures were prepared in a 10 to 1 and 3 to 1 effector to target (E:T) ratio in a U-bottom 96 well plate (Greiner, Vilvoorde, Belgium) with a total of 300.000 cells per well after which the plate was centrifuged for 1 min at 350 x g to increase interaction between effector and target cells. Co-cultures were incubated for 6 h after which viability of K562 target cells was assessed using Fixable Viability Dye eFluor 780 (FVS780, Thermo Fisher Scientific, Brussels, Belgium). K562 cells cultured in IMDM for 6 h in the absence of PBMCs was used as a negative control. K562 cells were discriminated from PBMCs based on FSC-A/SSC-A and subsequent CD7 and CD3 gating.

### Antibody dependent cellular cytotoxicity (ADCC) assay

PBMCs were thawed and rested overnight in complete IMDM, as described above. CEM.NKr CCR5^+^ cells were resuspended in Dulbecco’s phosphate-buffered saline (dPBS, VWR, Leuven, Belgium) at 1 × 10^6^ cells/mL after which 0.5 µg of recombinant gp120_BaL_ (obtained through the NIH HIV Reagent Program, Division of AIDS, NIAID, NIH: ARP-4961, contributed by DAIDS/NIAID; produced by ABLNIH AIDS reagent programme) was added. This mixture was incubated for 60 min at room temperature (RT). Excess rgp120 was subsequently washed off using dPBS. CEM.NKr CCR5^+^ cells were seeded in a U-bottom 96 well plate. Afterwards CD4 binding site antibody VRC01 (obtained through the NIH HIV Reagent Program, Division of AIDS, NIAID, NIH: Monoclonal Anti-HIV-1 gp120 Protein (VRC01, produced in vitro, ARP-12033, contributed by Dr. John Mascola) was added in a concentration of 1 µg/mL and incubated for 20 min at RT to allow binding to the gp120 coating. PBMCs were subsequently added in a 10 to 1 E:T ratio in 200 µL of complete IMDM medium with a total of 300.000 cells. As a control, Raji cells were co-cultured with PBMCs in the presence of 1 µg/mL Rituximab (Roche; Basel, Switzerland). Phorbol-12-myristate-13-acetate (PMA, Sigma-Aldrich; 10 ng/mL) with ionomycin (Sigma-Aldrich; 500 ng/mL) was used as a positive control for assessing cytokine production by NK cells. Next, 2 µL of anti-CD107a-BV421 (BioLegend, San Diego, CA, USA) was added to the wells. Finally, plates were centrifuged at 350 x g for 1 min and were incubated at 37 °C in a humidified 5% CO2 incubator for 12 h. After 1 h, Brefeldin A (BioLegend; 1/1000) and Monensin (BioLegend; 1/1000) were added to each well. NK cell (poly)functionality was assessed using intracellular cytokine staining, as described below. For each sample, some wells without Monensin, Brefeldin A and CD107a were prepared to assess NK cell-mediated killing of CEM.NKr CCR5^+^ cells using FVS780 staining. CEM.NKr CCR5^+^ cells were discriminated from PBMCs based on FSC-A/SSC-A and subsequent CD7 and CD3 gating. Percentage ADCC was calculated as follows:

$${{{\mathrm{\% }}}}ADCC = 100 \ast \frac{{(A - B)}}{{(C - B)}}$$

A = co-culture with antibody (experimental lysis), B = co-culture without antibody (spontaneous lysis) and C = maximal cell death of target cells in the presence of 20% EtOH.

### Flow cytometry

For phenotypic analysis of NK cells, PBMCs were thawed and immediately prepared for staining. Cells were first washed with dPBS and subsequently stained with FVS eFluor 780 (Thermo Fisher Scientific, Brussels, Belgium; 1/3500 dilution in dPBS) for 20 min at RT in the dark. Afterwards, the cells were stained with different antibody panels (3 × 10^6^ PBMCs/panel) for 30 min at 4 °C in the dark. Antibody dilutions were prepared in FACS buffer (dPBS containing 1% BSA (Sigma-Aldrich) and 0.1% sodium azide (Sigma-Aldrich)). All samples were stained with CD3, CD19 and CD14 to exclude T cells, B cells and monocytes respectively (DUMP panel) and with CD7, CD56 and CD16 (NK cell panel). CD7 was included to exclude CD56^+^ myeloid-derived cells as described by Milush et al*.*^59^. Additional antibodies were used for the different panels including KIR2DL1, KIR2DL2/3, KIR3DL1, CD94, NKp46 and NKG2D (NK cell receptor panel); CXCR5, CCR7 and CD62L (chemokine receptor panel); PD-1, LAG-3 and Tim3 (ICM panel); NKG2A, NKG2C, CD94 and CD57 (memory NK cell panel); and CD8 and CD30.

Polyfunctionality of NK cells was assessed by intracellular cytokine staining (ICS) using the Cyto-Fast^TM^ Fix/Perm buffer set (BioLegend), according to manufacturer’s protocol. The cells from the ADCC assay were harvested and subsequently stained with FVS780. Next, cells were stained with surface antibodies to identify NK cells (NK cell panel). Afterwards, cells were permeabilized using 250 µL of the CytoFast^TM^ Fix/Perm solution for 20 min at RT. Permeable cells were washed with CytoFast^TM^ Perm/Wash solution and stained for 30 min at 4 °C with antibodies against Perforin, IL-2, TNF-α, IFN-γ and MIP-1β diluted in CytoFast^TM^ Perm/Wash solution. Excess antibody was removed by washing with CytoFast^TM^ Perm/Wash solution and cells were resuspended in FACS buffer prior to acquisition on a BD LSR Fortessa (BD Biosciences, Erembodegem, Belgium). Compensation was performed using Compbeads (BD Biosciences) according to manufacturer’s protocol. Fluorescence minus one (FMO) controls were included to determine positive gates. A detailed overview of antibodies used for flow cytometry can be found in Supplementary Table 3.

### Data analysis and statistics

Flow cytometric analysis was performed using Flowlogic v7 (Inivai, Australia) and polyfunctionality was assessed using Boolean gating (Flowlogic v7). Multiparametric analysis was performed using Simplified Presentation of Incredibly Complex Evaluations (SPICE) (version 6.1, NIH, USA). Statistical analysis was performed using GraphPad Prism 9.4.1 software (San Diego, CA, USA). Data are presented as median ± interquartile range (IQR). Unless indicated otherwise in the figure legend, a Kruskal–Wallis test with Dunn’s multiple comparisons test was used for statistical comparison. Spearman rank correlation coefficients were calculated to determine the correlation between two continuous variables. *P*-values <0.05 were considered significant. The number of asterisks in the figures indicates the statistical significance as follows: *p < 0.05, **p < 0.005, ***p < 0.001, ****p < 0.0001.

### Reporting summary

Further information on research design is available in the Nature Research Reporting Summary linked to this article.

## Supplementary information

Supplementary Information

REPORTING SUMMARY

## Data Availability

All data generated during this study are included in the article and its Supplementary Information. The data that support the findings of the current study are available from the corresponding author upon request.
