# Supplementary material for: Autologous dendritic cell vaccination against HIV-1 induces changes in natural killer cell phenotype and functionality
Source: NPJ Vaccines. 2023 Mar 2;8:29. doi: 10.1038/s41541-023-00631-z (PMC9980861; doi:10.1038/s41541-023-00631-z)
Supplement: Supplementary file 1 — Supplementary Information [file 41541_2023_631_MOESM1_ESM.pdf]

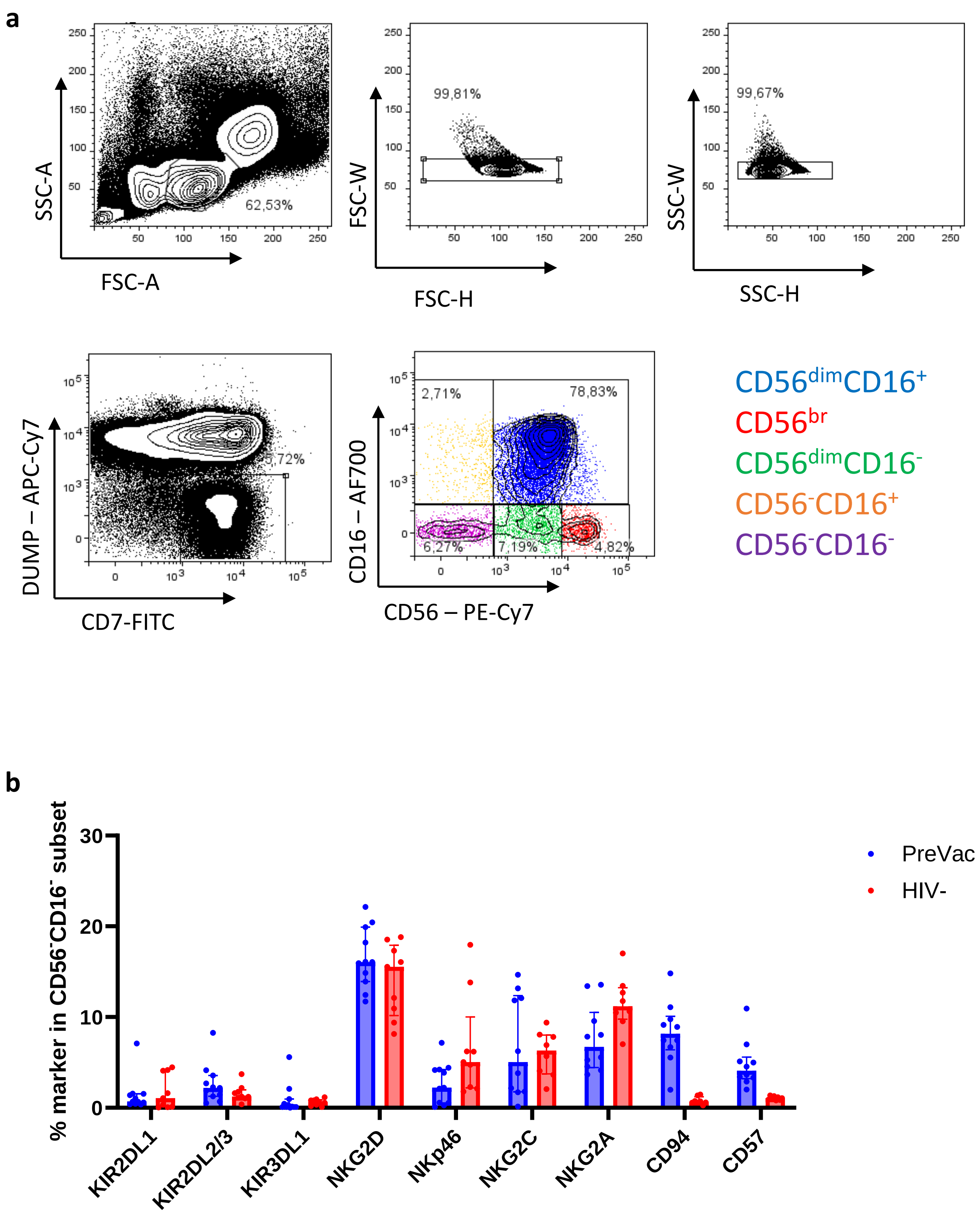

Supplementary Figure 1: Gating strategy human NK cells and phenotype of CD56$^-$CD16$^-$ cells

a) Complete gating strategy for identification of NK cells in PBMC samples. Gating on population of interest based on forward and side scatter (FSC-A and SSC-A) was followed by exclusion of cell aggregates. Preselection of NK cells using DUMP$^-$ (CD3, CD14, CD19 and LIVE/DEAD) and CD7$^+$ gating. Different NK cell subsets were identified using CD56 and CD16. **b)** Frequency of CD56$^-$CD16$^-$ NK cells expressing the indicated marker in HIV-1$^+$ (PreVac) and HIV-1$^-$ individuals. Data are presented as median ± IQR.

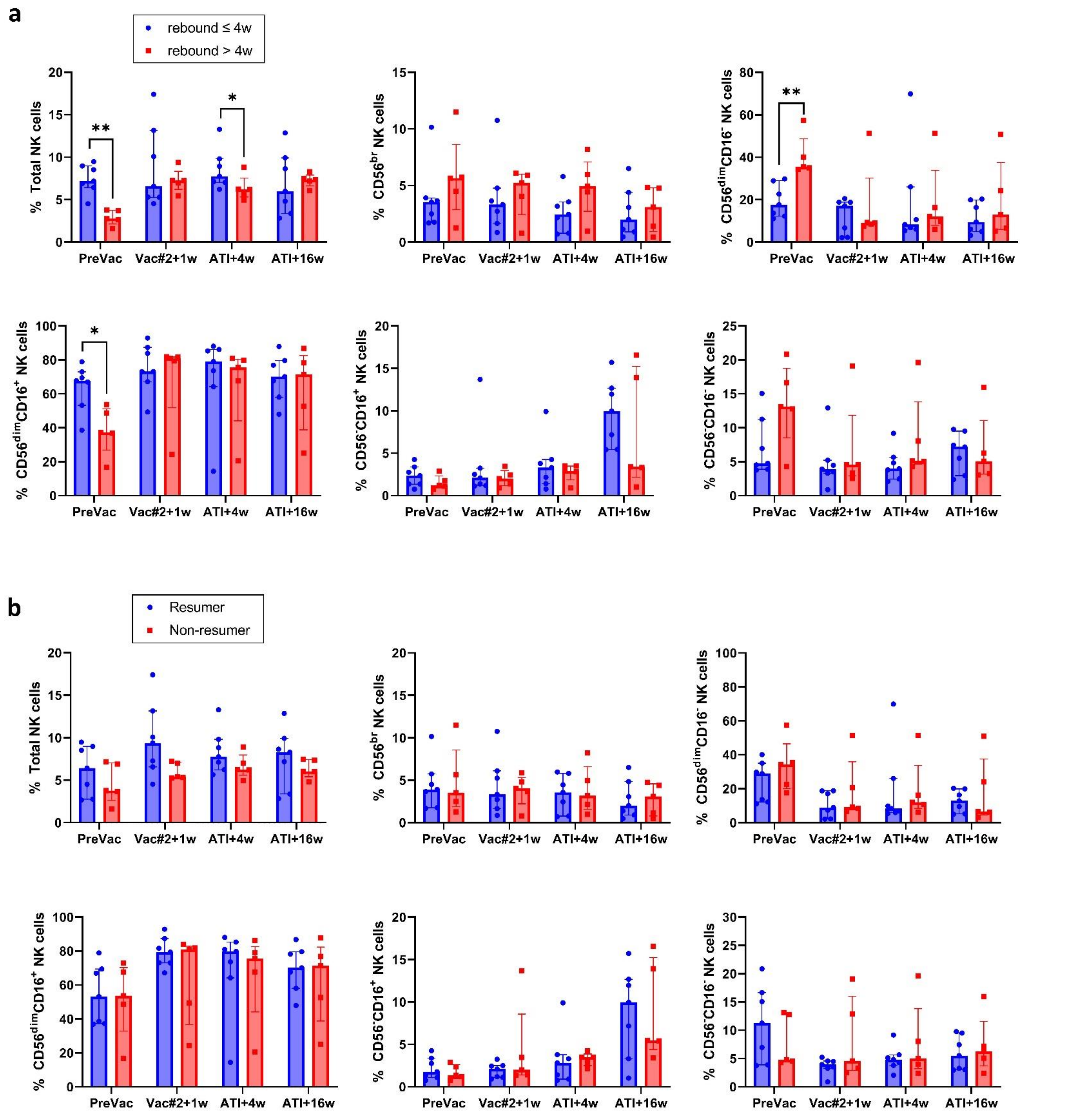

Supplementary Figure 2: NK cell frequency in resumers versus non-resumers and in early versus late rebounders

a) Comparison between the frequency of total NK cells and the different NK cell subsets in participants who rebounded before 4 weeks of ATI of after 4 weeks. **b)** Differences in total NK cell frequency and NK cell subsets between resumers (restart ART before 96 weeks of ATI) and non-resumers (restart ART after 96 weeks of ATI). Data are presented as median ± IQR, Mann-Whitney U test . P values < 0.05 were considered statistically significant and graphically annotated as follows: * $p < 0.05$ and ** $p < 0.005$.

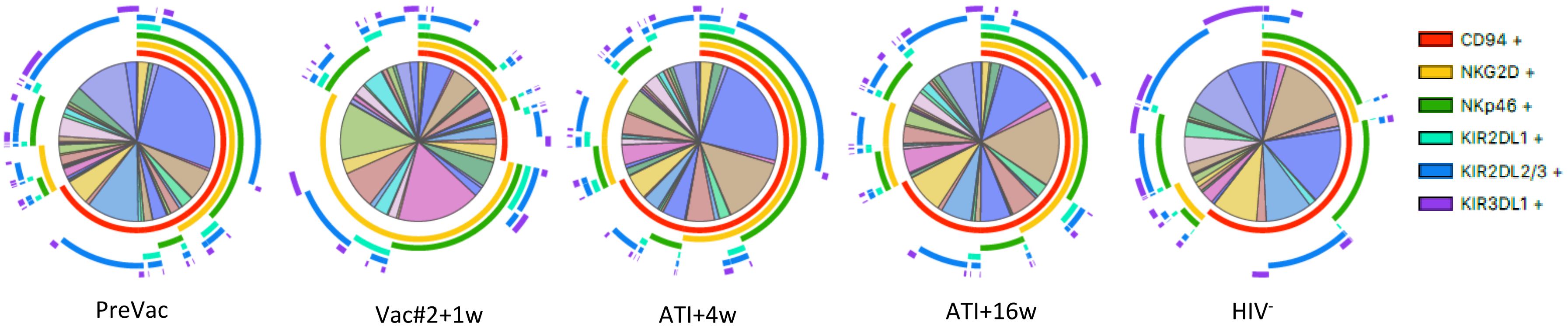

Supplementary Figure 3: Co-expression of NK cell receptors
SPICE analysis of NK cells co-expressing activating or inhibitory receptors.

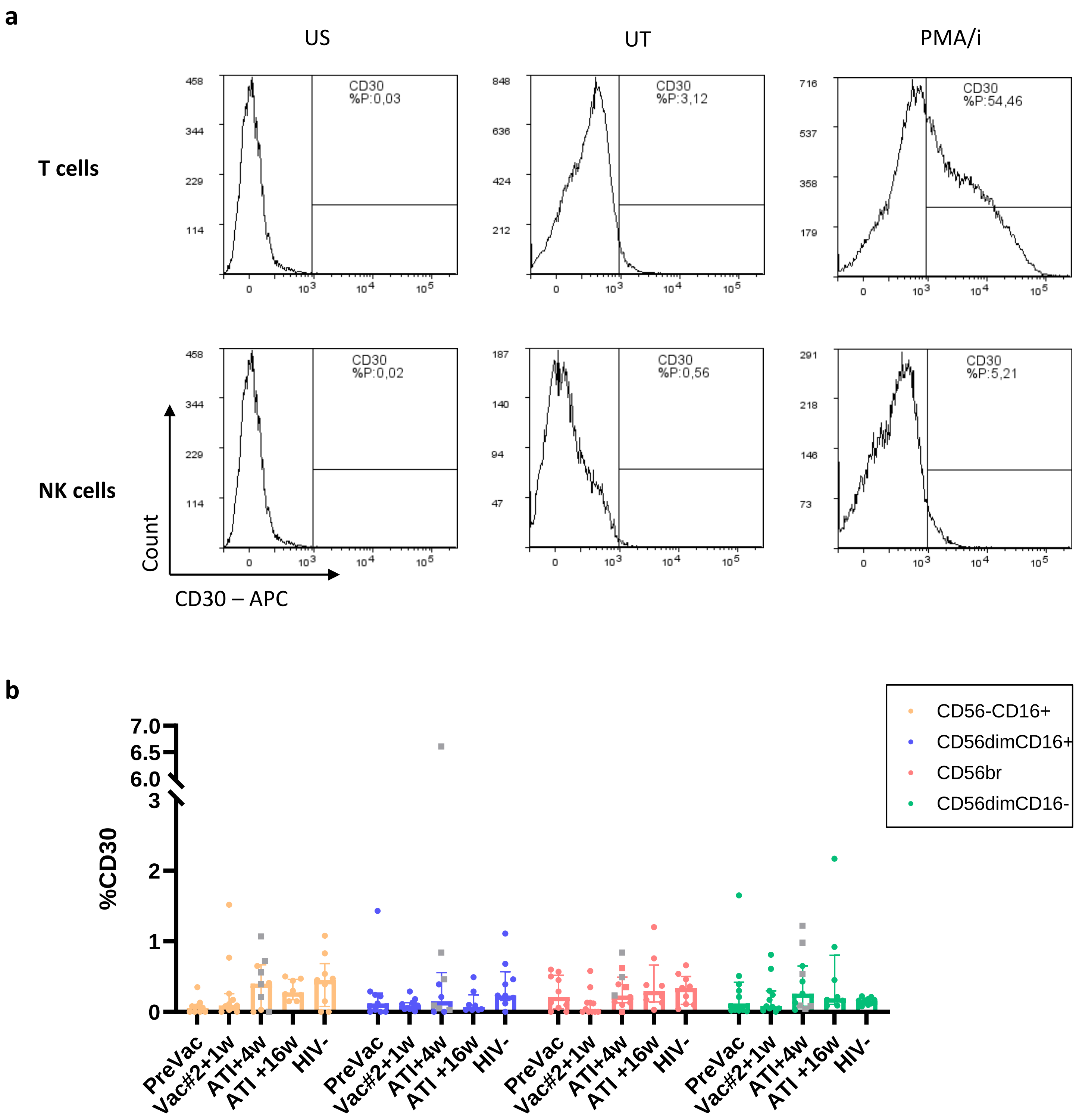

Supplementary Figure 4: Level of CD30 expressing NK cells

a) Gating for CD30 in unstained (US), untreated (UT) and PMA (10 ng/mL) + ionomycin (500 ng/mL) treated PBMCs. As a control, PBMCs were treated with PMA/I for 3 days and CD30 expression was checked on T cells (upper row) and NK cells (bottom row). **b)** Percentage of the different NK cell subsets expressing CD30 during the trial. Grey squares indicate participant who experienced viral rebound at ATI+4w. Median ± IQR, Kruskal-Wallis test with Dunn's multiple comparison test.

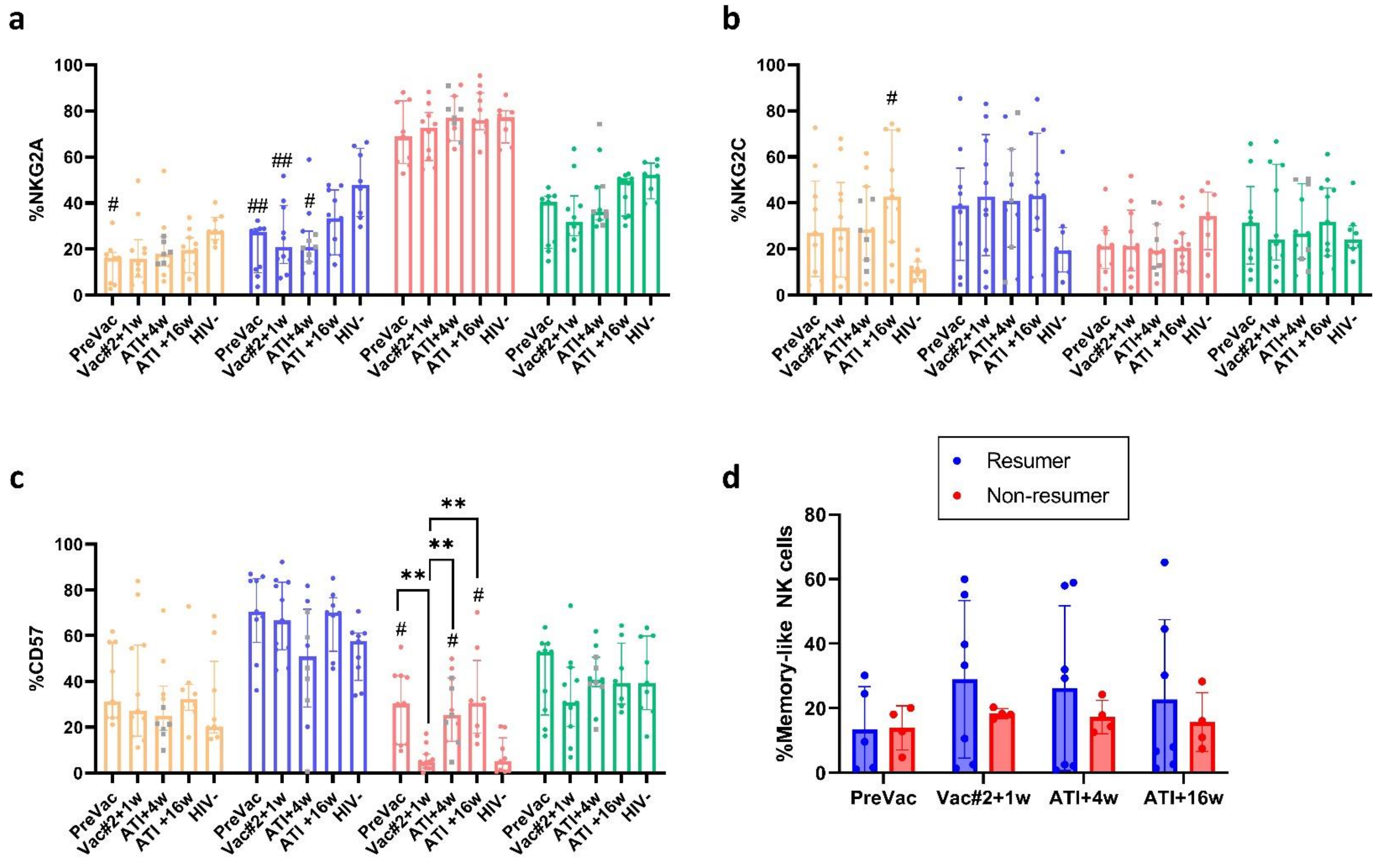

Supplementary Figure 5: Levels of NKG2A, NKG2C and CD57 expressing NK cells

a-c) Percentage of the different NK cell subsets expressing NKG2A, NKG2C or CD57 during the trial. Grey squares indicate participants who experienced viral rebound at ATI+4w. Median with IQR, Kruskal-Wallis with Dunn's multiple comparison. Significant differences between the studied time points are graphically annotated with '*'as follows: * $p < 0.05$, ** $p < 0.005$, *** $p < 0.001$ and **** $p < 0.0001$. '#' indicate a significant difference compared to HIV-1^{-} group. **d)** Comparison in percentage of memory-like NK cells between resumers and non-resumers.

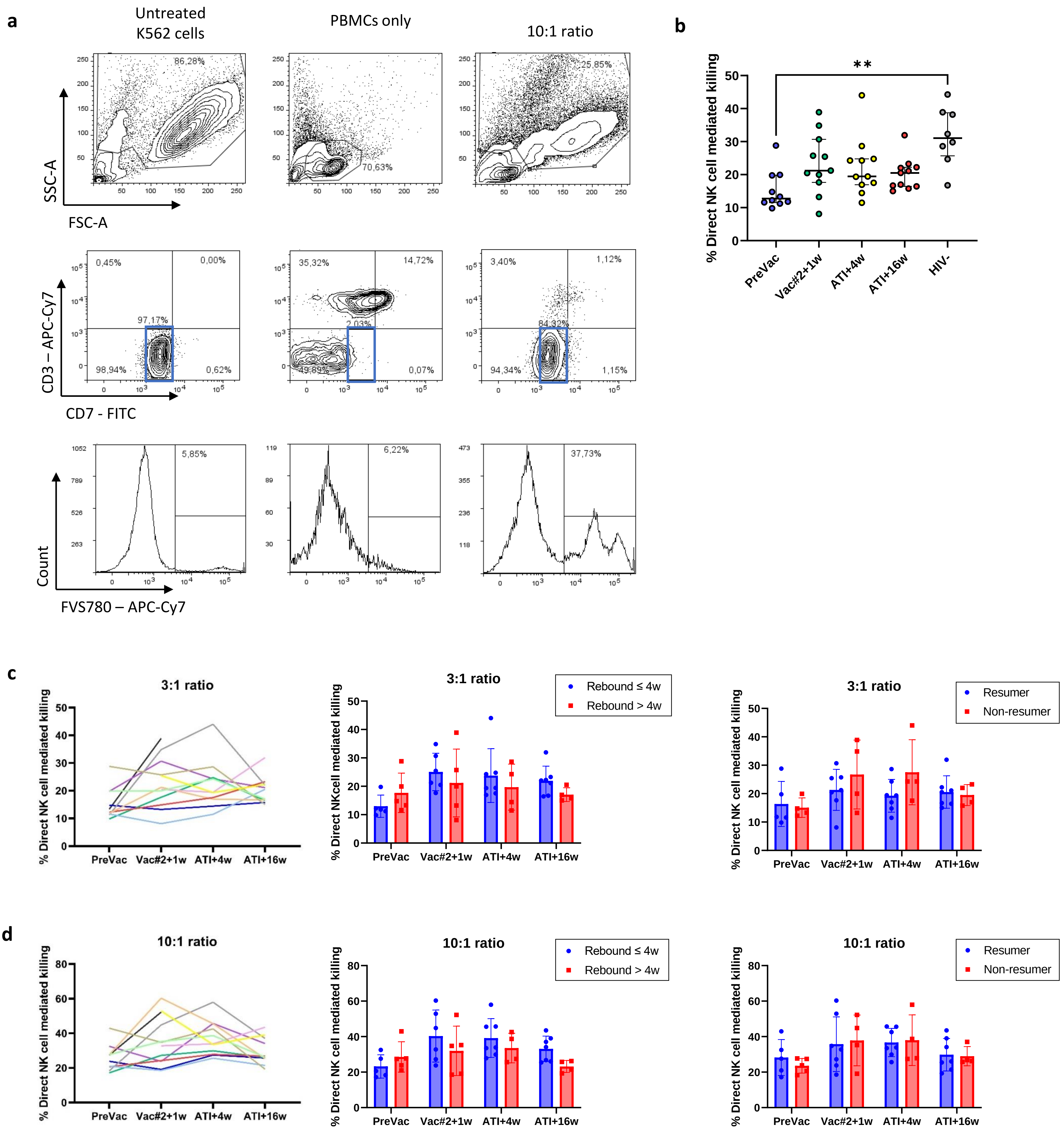

Supplementary Figure 6: Direct NK cell-mediated killing of K562 cells

a) Gating strategy for identification of viable and killed K562 cells (E:T of 10:1) using FVS780. **b)** Direct NK cell-mediated killing of K562 cells when co-cultured with PBMCs in 3:1 E:T ratio. Median with IQR, Kruskal-Wallis with Dunn's multiple comparison. **c)** Longitudinal analysis of NK cell-mediated cell death in individual participants (left). Comparison of NK cell-mediated cell death between participants that rebounded before or after 4 weeks of ATI (middle). Comparison of NK cell-mediated cell death between resumers and non-resumers. (right) for 3:1 effector to target ratio and **d)** 10:1 ratio. Data are presented as median with IQR; Mann-Whitney U test. Significant differences between the studied time points are graphically annotated with '*'as follows: ** $p < 0.005$.

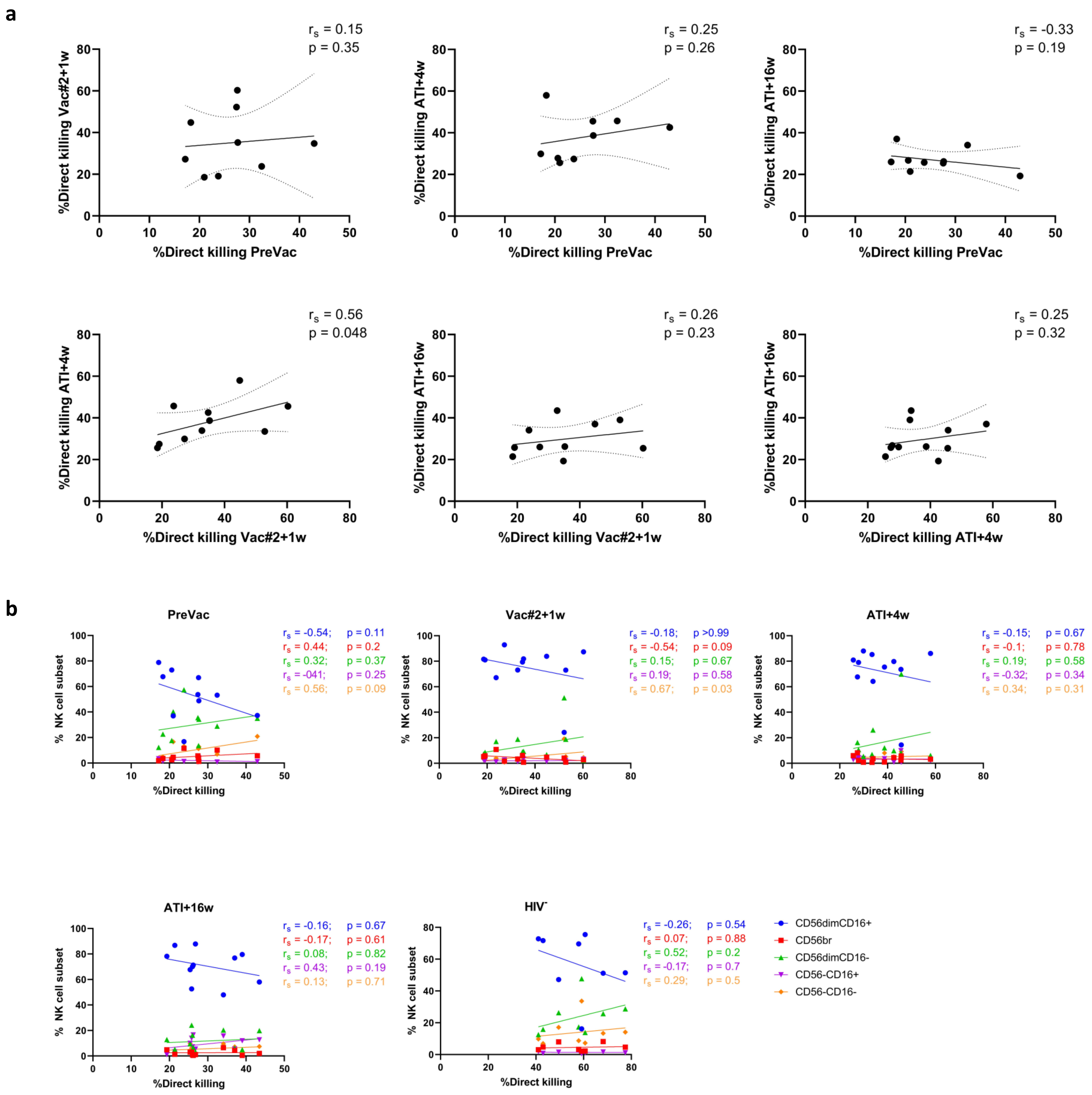

Supplementary Figure 7: Correlation of NK cell-mediated killing of K562 cells between the different time points of the trial and NK cell frequency

a) Correlation of the direct NK cell-mediated killing of K562 between the different time points of the trial. Spearman correlation coefficients and p values (two-sided) are indicated within the graphs. Dashed lines indicate 95% confidence interval. **b)** Correlation between the direct NK cell-mediated killing of K562 cells and the frequency of the NK cell subsets. Spearman correlation coefficients and p values (two-sided) are indicated within the graphs.

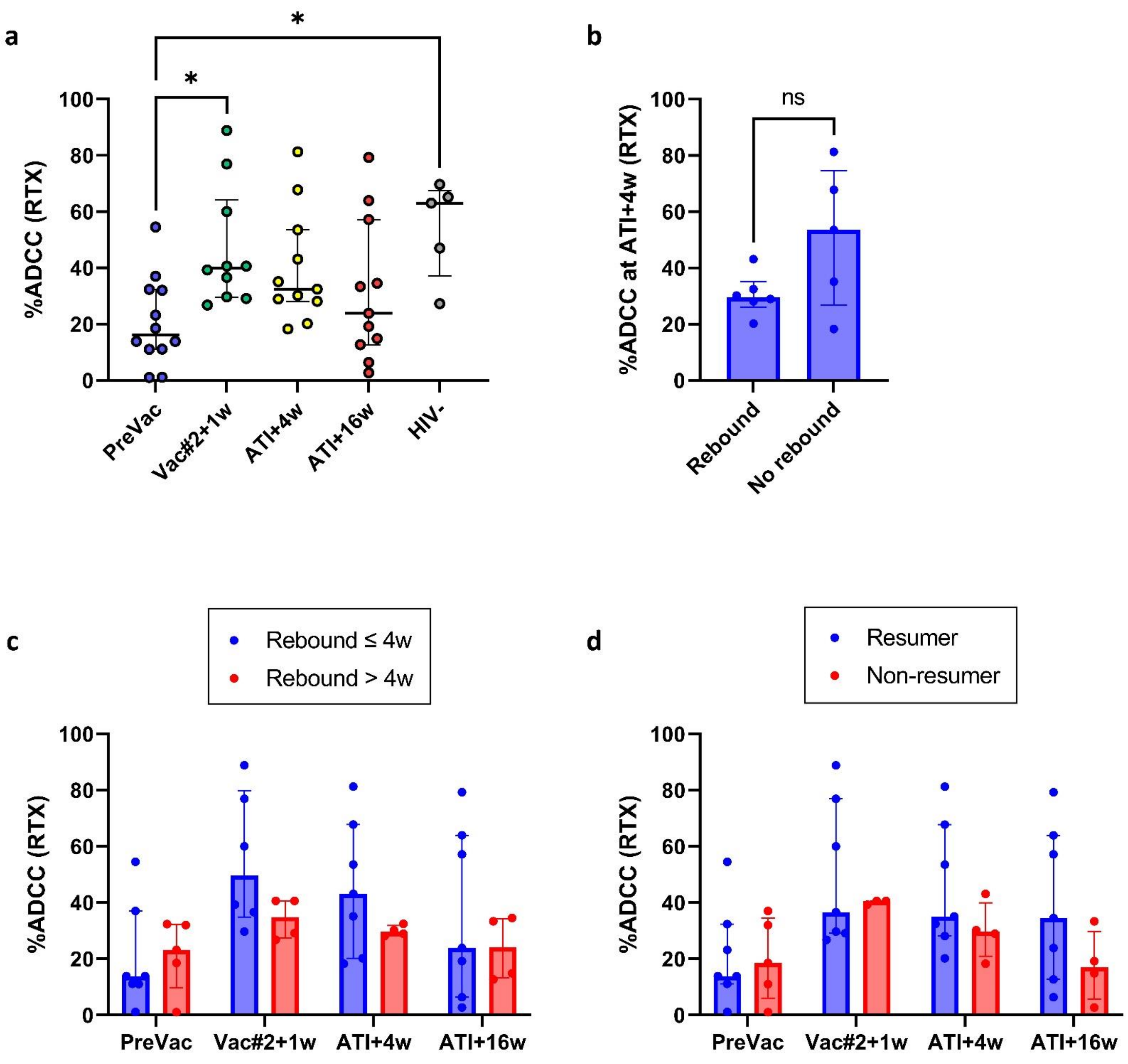

Supplementary Figure 8: ADCC-mediated killing of Raji cells

a) ADCC-mediated killing of Raji cells using Rituximab (RTX; 1 µg/mL) measured by flow cytometry (FVS780). Median with IQR, Kruskal-Wallis test with Dunn's multiple comparison test. **b)** Comparison of %ADCC at ATI+4w between participants that rebounded or not. **c)** Comparison of ADCC-mediated killing between participants that rebounded before or after 4 weeks of ATI. **d)** Comparison of ADCC-mediated cell death between resumers and non-resumers. Median with IQR, Mann-Whitney U test. *P* values < 0.05 were considered statistically significant and graphically annotated as follows: * $p < 0.05$.

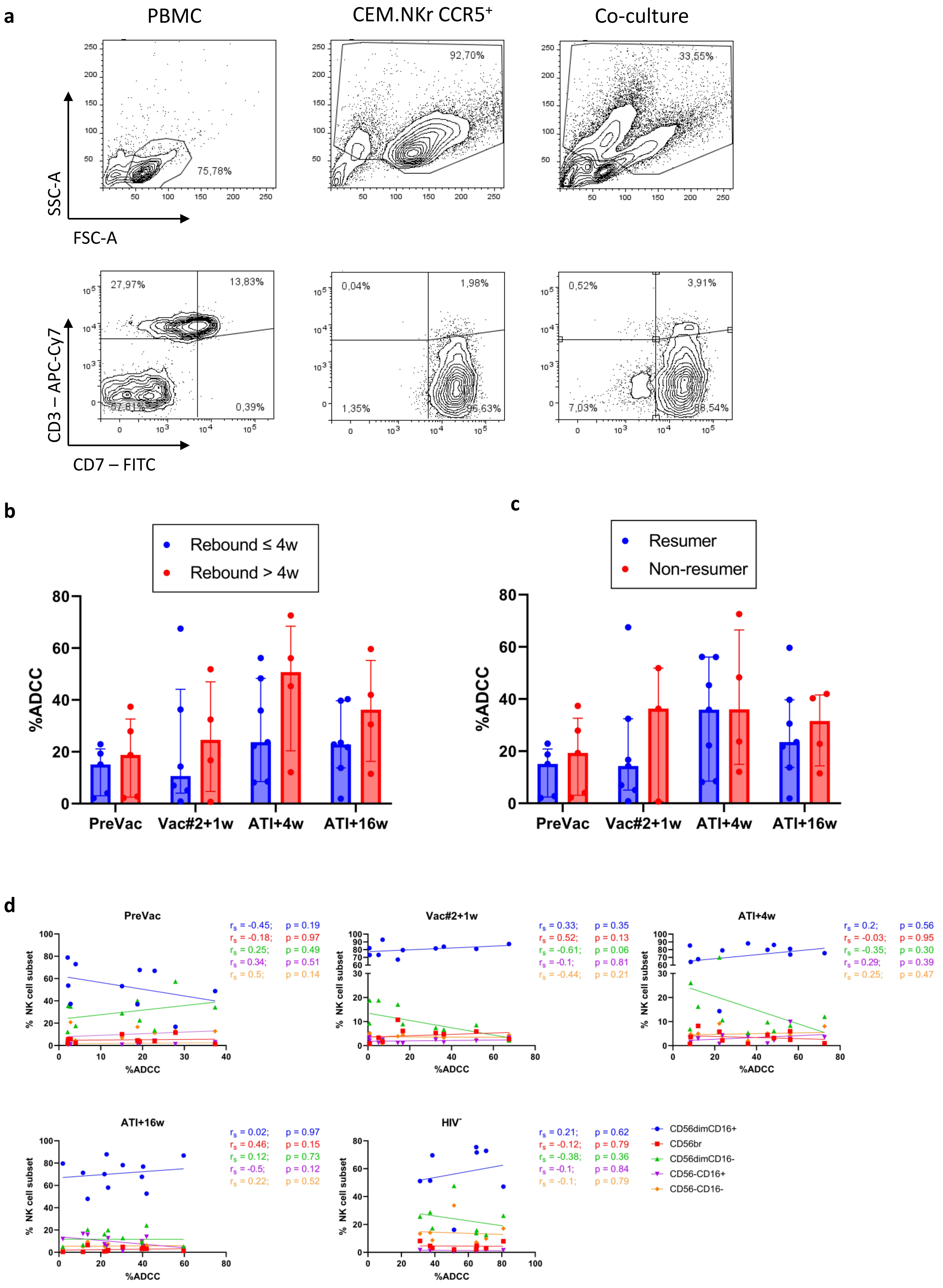

Supplementary Figure 9: ADCC-mediated cytotoxicity of CEM.NKr CCR5+ cells

a) Comparison of ADCC-mediated killing of CEM.NKr cells between participant who rebounded before or after 4 weeks of ATI. **b)** Comparison of ADCC-mediated killing of CEM.NKr cells between resumers and non-resumers. Median with IQR, Mann-Withney-U test. **c)** Correlation between ADCC-mediated killing and NK cells frequency. Spearman correlation coefficients and p values (two-sided) are indicated within the graphs.

a

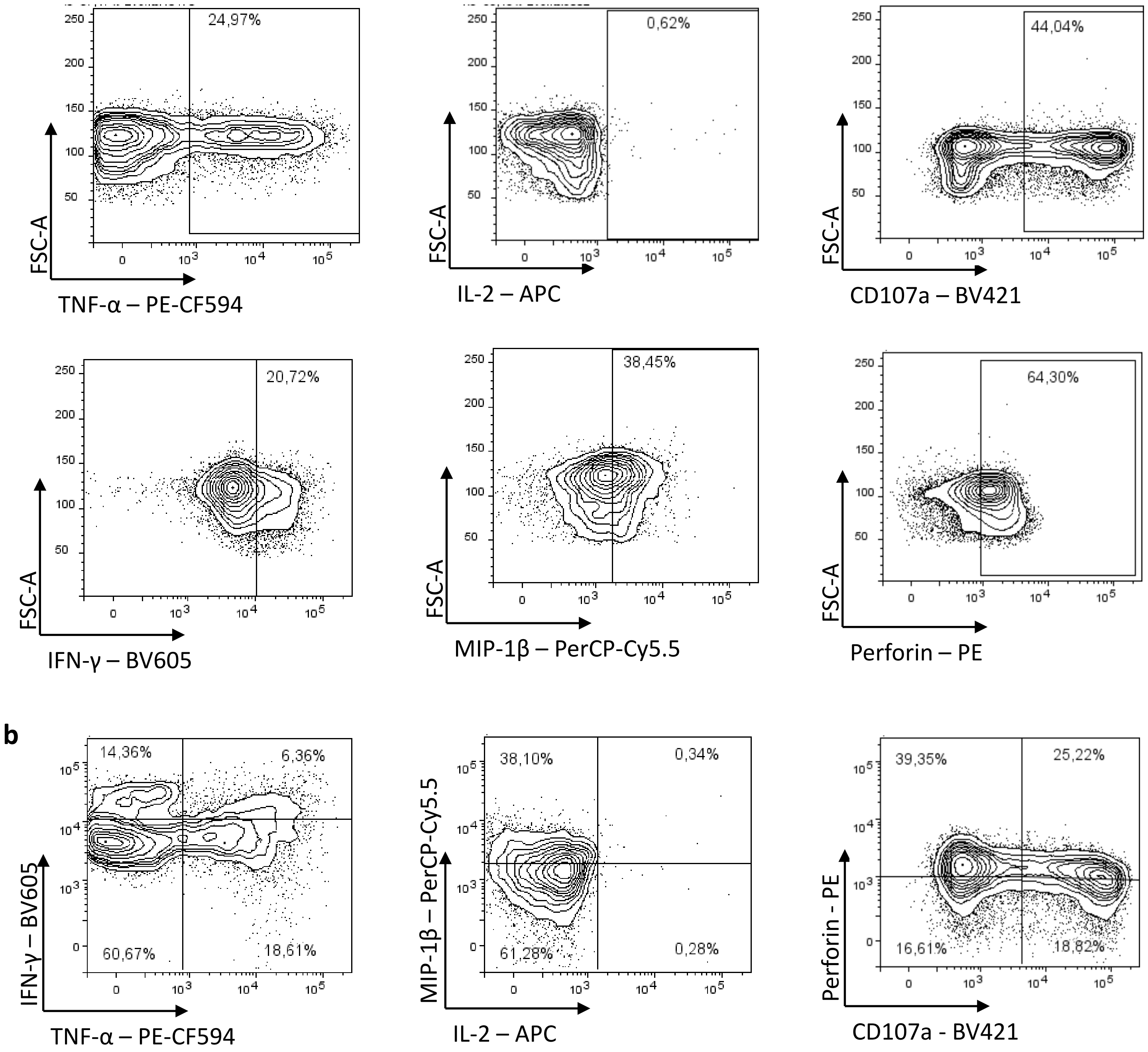

Supplementary Figure 10: Gating strategy for NK cell polyfunctionality

NK cells were identified as described in Supplementary Figure 1 and subsequent gating was performed on total NK cells that were stained for intracellular TNF-α, IFN-γ, IL-2, MIP-1β, CD107a and perforin in **a)** single plots and **b)** double plots.

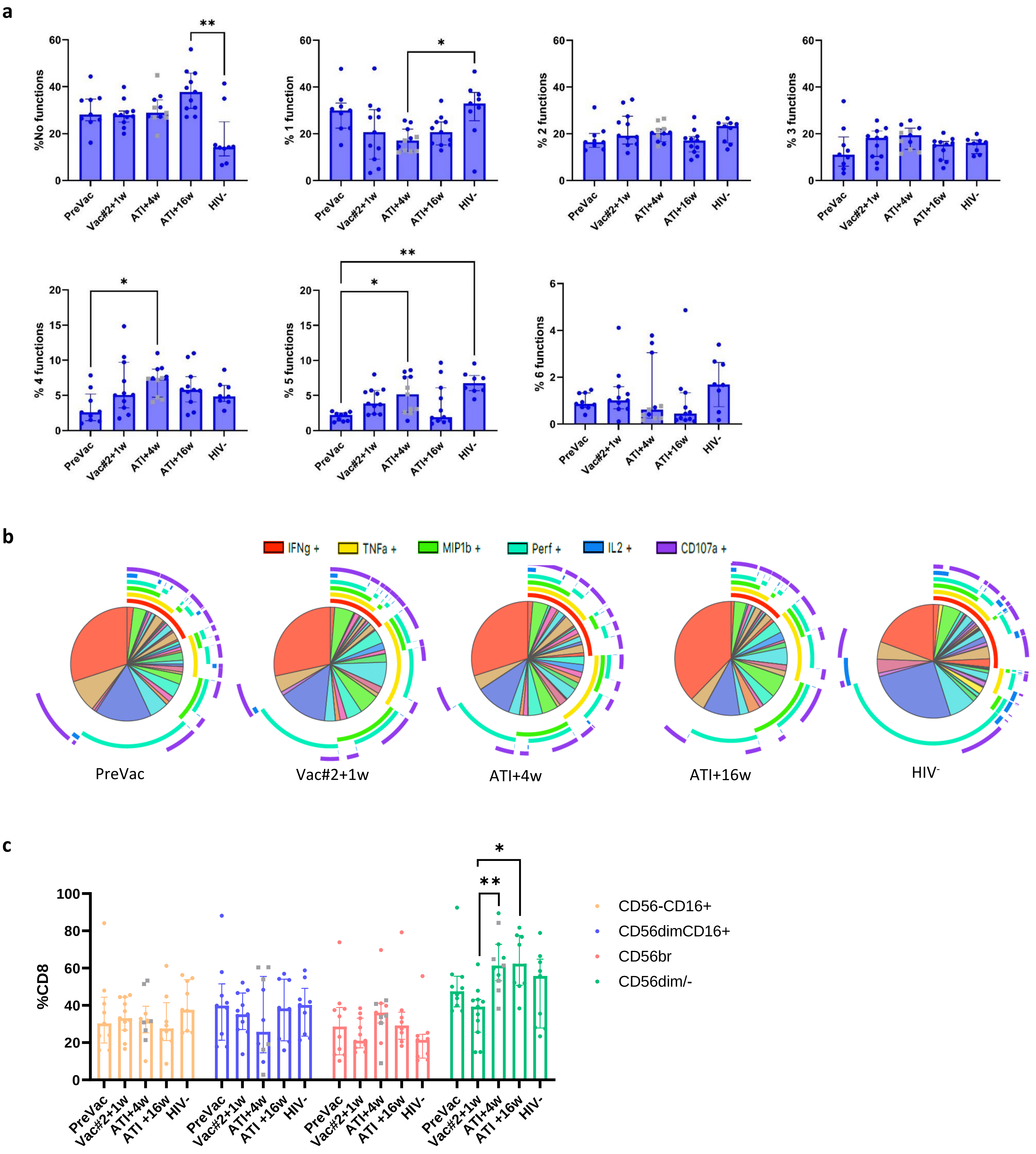

Supplementary Figure 11: NK cell polyfunctionality

a) Percentage of NK cells with 0-6 functions. Median with IQR, Kruskal-Wallis with Dunn's multiple comparison test. **b)** SPICE analysis for NK cell polyfunctionality at different timepoints of the study and in HIV-1$^-$ individuals. **c)** Percentage of NK cells subsets expressing CD8. Grey squares at ATI+4w indicate participant that have already rebounded. Squares indicate participant who experienced viral rebound at ATI+4w. Median with IQR, Kruskal-Wallis with Dunn's multiple comparison test. Significant differences between the studied time points are graphically annotated with '*'as follows: * $p < 0.05$ and ** $p < 0.005$

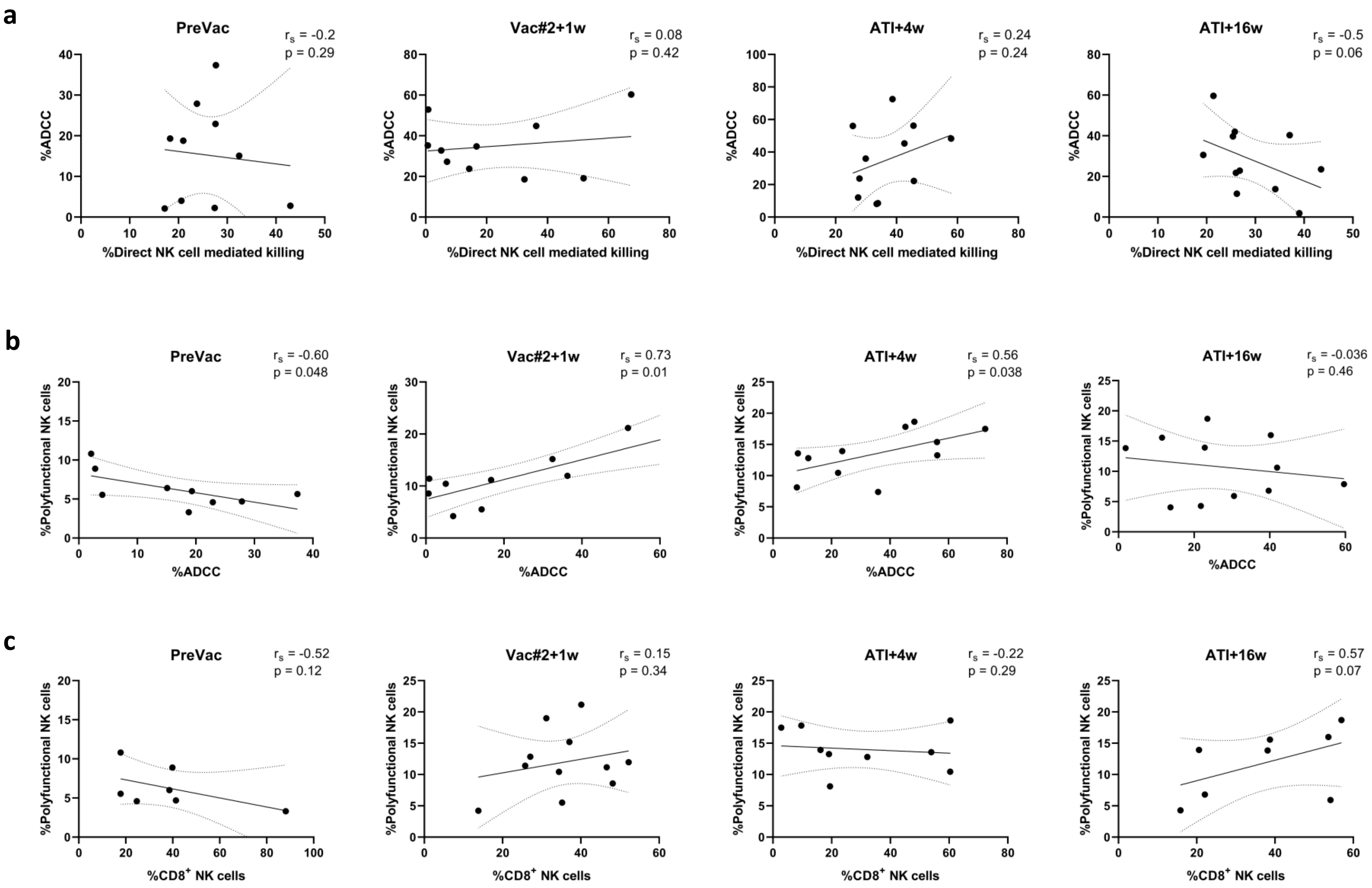

Supplementary Figure 12: Correlation NK cell functionality

a) Correlation between direct and indirect NK cell-mediated killing. **b)** Correlation between ADCC-mediated killing of CEM.NKr CCR5⁺ cells and NK cell polyfunctionality (defined as ≥4 functions). **c)** Correlation between percentage of CD8 expressing NK cells and polyfunctionality. Spearman correlation coefficients and p values (two-sided) are indicated within the graphs. Dashed lines indicate 95% confidence interval.

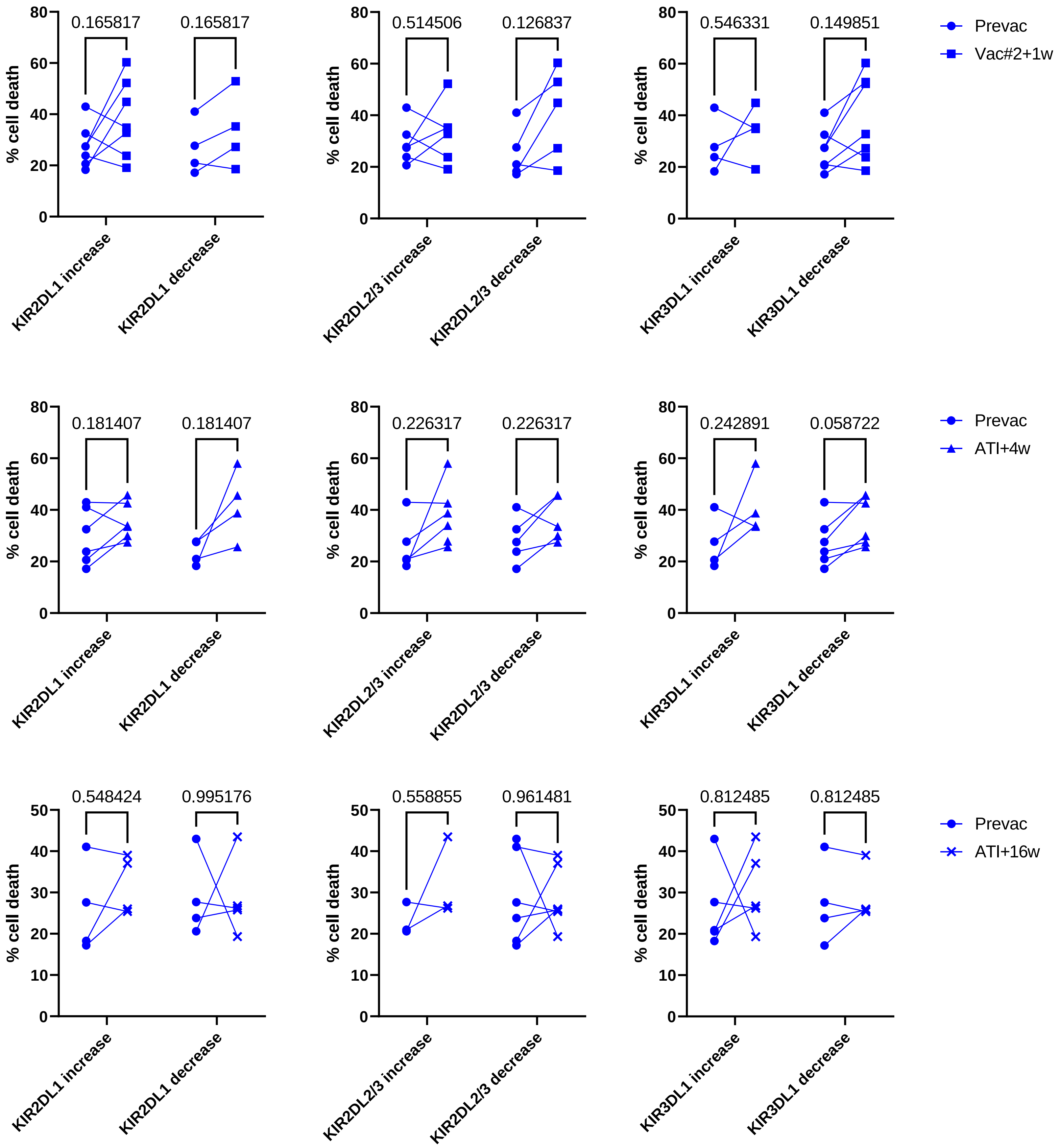

Supplementary Figure 13: Link between KIR expression and direct NK cell-mediated killing
Participants were subdivided based on an increase or decrease in NK cells expressing the indicated KIRs between PreVac and Vac#2+1w or PreVac and ATI+4w or PreVac and ATI+16w. Direct NK cell-mediated cell death (K562 cells) was compared between these groups. Paired t-test was performed and p values (two-sided) are indicated within the graphs.

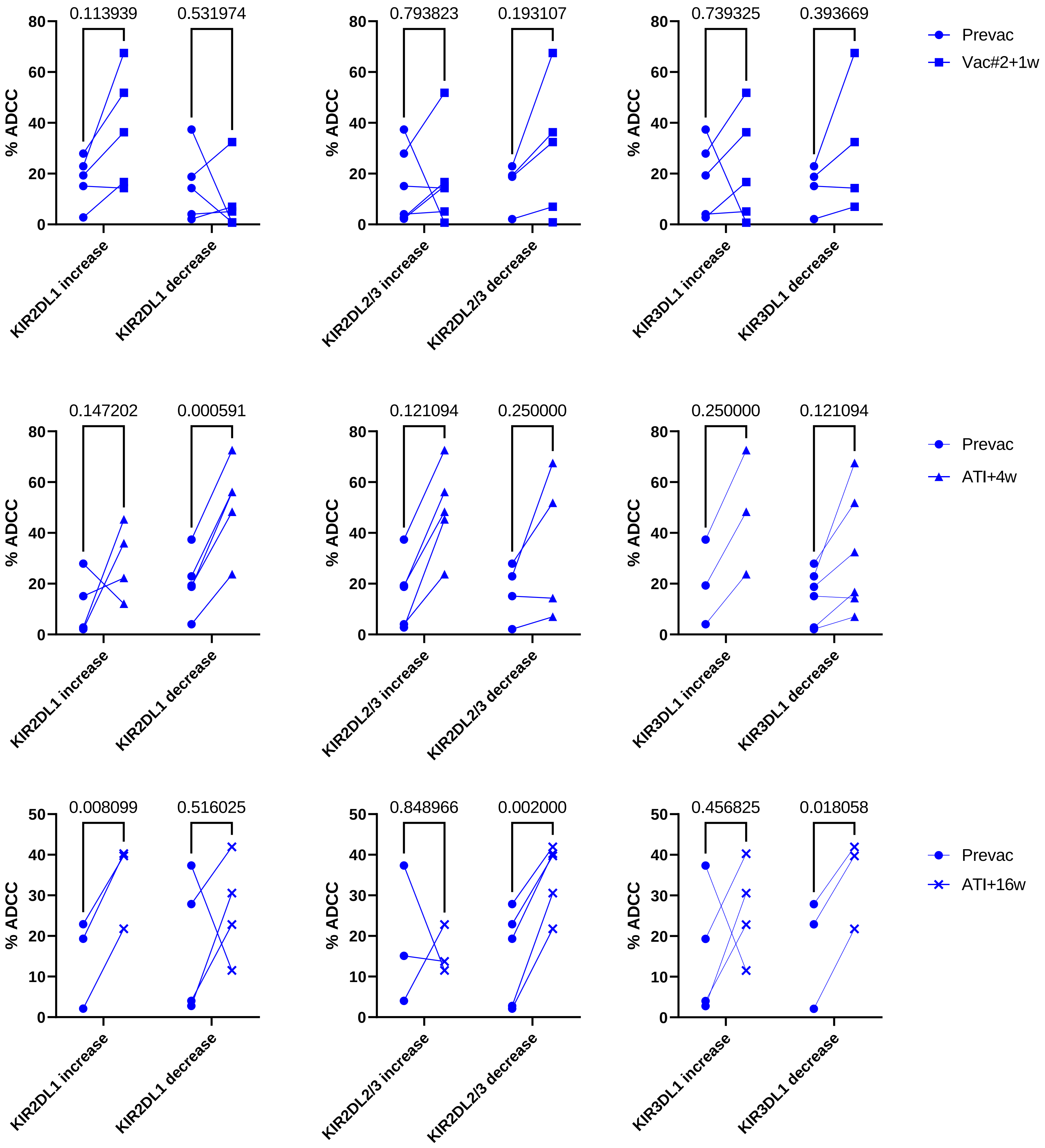

Supplementary Figure 14: Link between KIR expression and ADCC-mediated killing of CEM.NKr CCR5^{+} cells
Participants were subdivided based on an increase or decrease in NK cells expressing the indicated KIRs between PreVac and Vac#2+1w or PreVac and ATI+4w or PreVac and ATI+16w. ADCC-mediated cell death of CEM.NKr CCR5^{+} cells was compared between these groups. Paired t-test (two-sided) was performed and p values are indicated within the graphs.

PARTICIPANT	HLA-A	HLA-B	HLA-C	%KIR2DL1 PREVAC	%KIR2DL2/3 PREVAC	%KIR3DL1 PREVAC
**H001**	0301	0702	07	2.27	4.93	5.66
	2902	4403	16			
**H002**	0201	2705	01	4.81	16.22	4.24
	3201	3801	12			
**H003**	0201	4002	02	8.04	11.2	2.30
	2902	4402	05			
**H004**	0101	0801	03	40.11	4.59	2.89
	2402	4001	07			
**H005**	2402	3501	04	39.78	4.48	1.83
	3201	4402	05			
**H006**	0101	0702	07	6.24	3.18	1.89
	2402	0801				
**H007**	0201	0702	07	4.24	24.39	22.82
	0301	4402				
**H008**	0201	1302	06	13.39	40.95	14.21
	1101	5701				
**H014**	0101	0702	0702	5.31	7.44	5.68
	0201					
**H015**	0201	1501	0304	22.57	22.6	7.05
	0201	5101	1502			
**H016**	3101	4001	0202	10.75	12.34	4.61
	3201	4002	0304			
**H017**	0301	3501	0401	19.95	52.58	8.74
	2901	5101	1402			

Supplementary Table 1: HLA genotyping of DC-TRN study participants

For each participant, HLA-A/B/C genotyping was performed. Colours indicate the corresponding KIR receptors (KIR2DL1 in green, KIR2DL2/3 in blue, KIR3DL1 in red). The percentage of NK cells expressing KIR2SL1, KIR2DL2/3 or KIR3DL1 are given for all participants before vaccination (PreVac).

CHARACTERISTICS	DC-TRN GROUP (N = 12)	HIV-1^{-} GROUP (N = 8)
AGE (YEARS)	43.5 (36-56)	26.5 (22-43)
GENDER (MALE)	100%	50%
YEAR OF HIV-1 DIAGNOSIS	1997 (1992-2004)	-
TIME ON ART BEFORE ENROLLMENT (WEEKS)	113.5 (36-142)	-
TIME BETWEEN HIV-1 DIAGNOSIS AND ART INITIATION (WEEKS)	31 (2-442)	-
TIME UNTIL HIV-1 RNA > 3 LOG_{10} COPIES/ML FOLLOWING ATI (WEEKS)	4 (2-8)	-
WEEKS OFF ART (WEEKS)	51 (28-352)	-
NADIR $CD4^{+}$ T CELLS (/MM^{3})	360 (290-650)	-

Supplementary Table 2: Characteristics of DC-TRN study participants and HIV-1^{-} donors

Results are shown as median with range. Age and time on ART at the first immunisation are given.

TARGET	FLUOROPHORE	CLONE	CATALOG N°	LOT N°	SUPPLIER
**CD3**	APC-Cy7	SK7	557832	1224177	BD Biosciences
**CD14**	APC-Cy7	MϕP9	557831	7024588	BD Biosciences
**CD19**	APC-Cy7	SJ25C1	557791	9064665	BD Biosciences
**CD7**	FITC	M-T701	565211	1277599	BD Biosciences
**CD56**	PE-Cy7	B159	557747	1326783	BD Biosciences
**CD16**	AlexaFluor700	3G8	560713	2010575	BD Biosciences
**KIR2DL1**	BV421	HP-3E4	564318	8064803	BD Biosciences
**KIR2DL2/3**	BV510	CH-L	743452	7082701	BD Biosciences
**KIR3DL1**	BV605	DX9	742981	7082941	BD Biosciences
**NKG2D**	PerCP-Cy5.5	1D11	320818	B249077	BioLegend
**NKp46**	APC	9E2	558051	8151558	BD Biosciences
**CD94**	PE	HP 3D9	555889	6140799	BD Biosciences
**NKG2A**	BV421	131411	747924	1019334	BD Biosciences
**NKG2C**	APC	REA205	130-117-398	5220500831	Miltenyi Biotec
**CD57**	PE/Dazzle	HNK-1	359620	B300705	BioLegend
**PD-1**	PE	EH12.2H7	329906	B214406	BioLegend
**LAG-3**	BV421	T47-530	565720	8087744	BD Biosciences
**TIM3**	PerCP-Cy5.5	F38-2E2	345016	B292773	BioLegend
**CXCR5**	BV421	RF8B2	562747	8025974	BD Biosciences
**CCR7**	AlexaFluor647	3D12	557734	7215908	BD Biosciences
**CD62L**	PE	DREG	565411	7061971	BD Biosciences
**CD8**	BV510	SK1	563919	7201643	BD Biosciences
**CD30**	APC	BerH8	563500	7187726	BD Biosciences
**PERFORIN**	PE	BD48	854.952.010	E120611	Diaclone
**CD107a**	BV421	H4A3	328626	B354870	BioLegend
**TNF-α**	PE-CF594	mAb11	562784	9171775	BD Biosciences
**IL-2**	APC	MQ1-17H12	17-7029-82	4303346	eBiosciences
**IFN-γ**	BV605	B27	562974	9176973	BD Biosciences
**MIP-1β**	PerCP-Cy5.5	FL34Z3L	46-7540-42	2480322	eBiosciences

Supplementary Table 3: Overview of antibodies that were used for flow cytometry.

For each antibody, the fluorophore, clone, catalogue number, lot number and supplier are provided.
