# Supplementary material for: Autologous dendritic cell vaccination against HIV-1 induces changes in natural killer cell phenotype and functionality
Source: NPJ Vaccines. 2023 Mar 2;8:29. doi: 10.1038/s41541-023-00631-z (PMC9980861; doi:10.1038/s41541-023-00631-z)
Supplement: Supplementary file 2 — REPORTING SUMMARY [file 41541_2023_631_MOESM2_ESM.pdf]

nature portfolio

Corresponding author(s): Joeri Aerts

Last updated by author(s): 2023/01/11

Reporting Summary

Nature Portfolio wishes to improve the reproducibility of the work that we publish. This form provides structure for consistency and transparency in reporting. For further information on Nature Portfolio policies, see our Editorial Policies and the Editorial Policy Checklist.

Statistics

For all statistical analyses, confirm that the following items are present in the figure legend, table legend, main text, or Methods section.

n/a	Confirmed	
☐	☒	The exact sample size (*n*) for each experimental group/condition, given as a discrete number and unit of measurement
☐	☒	A statement on whether measurements were taken from distinct samples or whether the same sample was measured repeatedly
☐	☒	The statistical test(s) used AND whether they are one- or two-sided<br>*Only common tests should be described solely by name; describe more complex techniques in the Methods section.*
☒	☐	A description of all covariates tested
☐	☒	A description of any assumptions or corrections, such as tests of normality and adjustment for multiple comparisons
☐	☒	A full description of the statistical parameters including central tendency (e.g. means) or other basic estimates (e.g. regression coefficient) AND variation (e.g. standard deviation) or associated estimates of uncertainty (e.g. confidence intervals)
☐	☒	For null hypothesis testing, the test statistic (e.g. *F*, *t*, *r*) with confidence intervals, effect sizes, degrees of freedom and *P* value noted<br>*Give P values as exact values whenever suitable.*
☒	☐	For Bayesian analysis, information on the choice of priors and Markov chain Monte Carlo settings
☒	☐	For hierarchical and complex designs, identification of the appropriate level for tests and full reporting of outcomes
☐	☒	Estimates of effect sizes (e.g. Cohen's *d*, Pearson's *r*), indicating how they were calculated

Our web collection on statistics for biologists contains articles on many of the points above.

Software and code

Policy information about availability of computer code

Data collection	BD LSR Fortessa (FACS Diva software) was used to collect flow cytometry data.
Data analysis	All data analysis was performed using GraphPad Prism (version 9.4.1). Flow cytometry data was analysed using Flowlogic v7 and polyfunctionality was assessed using SPICE (version 6.1).

For manuscripts utilizing custom algorithms or software that are central to the research but not yet described in published literature, software must be made available to editors and reviewers. We strongly encourage code deposition in a community repository (e.g. GitHub). See the Nature Portfolio guidelines for submitting code & software for further information.

Data

Policy information about availability of data

All manuscripts must include a data availability statement. This statement should provide the following information, where applicable:

- Accession codes, unique identifiers, or web links for publicly available datasets
- A description of any restrictions on data availability
- For clinical datasets or third party data, please ensure that the statement adheres to our policy

All data generated during this study are included in the article and its supplementary information. The data that support the findings of the current study are available from the corresponding author upon request.

Human research participants

Policy information about studies involving human research participants and Sex and Gender in Research.

Reporting on sex and gender	All participants in the vaccination group were males, whereas both genders were equally present in the HIV-1 uninfected control group.
Population characteristics	Population characteristics are provided in Supplementary table 2.
Recruitment	Participants were recruited from the PLWH in care at the medical centers, by the treating physician
Ethics oversight	The study was approved by the institutional boards of UZ Brussel (Belgium) and Erasmus Medical Center (The Netherlands).

Note that full information on the approval of the study protocol must also be provided in the manuscript.

Field-specific reporting

Please select the one below that is the best fit for your research. If you are not sure, read the appropriate sections before making your selection.

☒ Life sciences ☐ Behavioural & social sciences ☐ Ecological, evolutionary & environmental sciences

For a reference copy of the document with all sections, see nature.com/documents/nr-reporting-summary-flat.pdf

Life sciences study design

All studies must disclose on these points even when the disclosure is negative.

Sample size	As this is a Phase I/IIa study, no sample size calculation was performed.
Data exclusions	No data was excluded from the analysis. Missing values are due to limited availability of biological material of due to limited viability of the samples upon thawing.
Replication	No replicates were used.
Randomization	Not applicable. This study has only one treatment group.
Blinding	Investigators were blinded during data analysis.

Reporting for specific materials, systems and methods

We require information from authors about some types of materials, experimental systems and methods used in many studies. Here, indicate whether each material, system or method listed is relevant to your study. If you are not sure if a list item applies to your research, read the appropriate section before selecting a response.

Materials & experimental systems

n/a	Involved in the study	
☐	☒	Antibodies
☐	☒	Eukaryotic cell lines
☒	☐	Palaeontology and archaeology
☒	☐	Animals and other organisms
☐	☒	Clinical data
☒	☐	Dual use research of concern

Methods

n/a	Involved in the study	
☒	☐	ChIP-seq
☐	☒	Flow cytometry
☒	☐	MRI-based neuroimaging

Antibodies

Antibodies used	All information is provided in Supplementary table 3.
Validation	Antibodies were validated by the manufacturer.

Eukaryotic cell lines

Policy information about cell lines and Sex and Gender in Research

Cell line source(s)	CEM.NKr CCR5+ cells (kindly provided by Carole Devaux, LIH and originally purchased from AIDS reagents program). K562 cells (kindly provided by Karine Brekpot, VUB and originally purchased from ATCC
Authentication	Authentication was performed by AIDS reagent program and ATCC.
Mycoplasma contamination	All cell lines are regulary tested for Mycoplasma (Mycoplasma Detection Kit; Invivogen). Both cell lines used in this study were mycoplasma-free.
Commonly misidentified lines (See ICLAC register)	Not applicable.

Clinical data

Policy information about clinical studies

All manuscripts should comply with the ICMJE guidelines for publication of clinical research and a completed CONSORT checklist must be included with all submissions.

Clinical trial registration	NTR2198 (https://trialsearch.who.int/Trial2.aspx?TrialID=NTR2198)
Study protocol	Upon request from the authors
Data collection	Participants were included in Brussels and Rotterdam, between November 8, 2006 (first screening first patient) and August 10, 2008, (last participant, last visit)
Outcomes	Safety, tolerability and immunogenicity are described in manuscript Allard et al (AIDS 2012) PMID: 22177848

Flow Cytometry

Plots

Confirm that:

☒ The axis labels state the marker and fluorochrome used (e.g. CD4-FITC).

☒ The axis scales are clearly visible. Include numbers along axes only for bottom left plot of group (a 'group' is an analysis of identical markers).

☒ All plots are contour plots with outliers or pseudocolor plots.

☒ A numerical value for number of cells or percentage (with statistics) is provided.

Methodology

Sample preparation	PBMCs were isolated from whole blood and were stored in liquid nitrogen until further use.
Instrument	BD LSR Fortessa
Software	For data collection, FACS Diva (v7) was used. Flowlogic v7 was used to analyse the data.
Cell population abundance	No sorting was performed in this study.
Gating strategy	Gataing strategy is shown in Supplementary figures.

☒ Tick this box to confirm that a figure exemplifying the gating strategy is provided in the Supplementary Information.
